# Two-Dimensional MoS_2_ Nanosheets Derived from Cathodic Exfoliation for Lithium Storage Applications

**DOI:** 10.3390/nano14110932

**Published:** 2024-05-25

**Authors:** Alberto Martínez-Jódar, Silvia Villar-Rodil, José M. Munuera, Alberto Castro-Muñiz, Jonathan N. Coleman, Encarnación Raymundo-Piñero, Juan I. Paredes

**Affiliations:** 1 Instituto de Ciencia y Tecnología del Carbono, INCAR-CSIC, Francisco Pintado Fe 26, 33011 Oviedo, Spain; alberto.mj@incar.csic.es (A.M.-J.); alberto@incar.csic.es (A.C.-M.); 2 CEMHTI UPR3079, University of Orléans, CNRS, 1D avenue de la Recherche Scientifique, 45071 Orléans, France; encarnacion.raymundo@cnrs-orleans.fr; 3 Department of Physics, Faculty of Sciences, University of Oviedo, C/ Leopoldo Calvo Sotelo, 18, 33007 Oviedo, Spain; munuerajose@uniovi.es; 4 School of Physics, CRANN and AMBER Research Centre, Trinity College Dublin, D02 E8C0 Dublin, Ireland; colemaj@tcd.ie

**Keywords:** transition metal dichalcogenide (TMD), MoS_2_, electrochemical exfoliation, energy storage, lithium-ion batteries

## Abstract

The preparation of 2H-phase MoS_2_ thin nanosheets by electrochemical delamination remains a challenge, despite numerous efforts in this direction. In this work, by choosing appropriate intercalating cations for cathodic delamination, the insertion process was facilitated, leading to a higher degree of exfoliation while maintaining the original 2H-phase of the starting bulk MoS_2_ material. Specifically, trimethylalkylammonium cations were tested as electrolytes, outperforming their bulkier tetraalkylammonium counterparts, which have been the focus of past studies. The performance of novel electrochemically derived 2H-phase MoS_2_ nanosheets as electrode material for electrochemical energy storage in lithium-ion batteries was investigated. The lower thickness and thus higher flexibility of cathodically exfoliated MoS_2_ promoted better electrochemical performance compared to liquid-phase and ultrasonically assisted exfoliated MoS_2_, both in terms of capacity (447 vs. 371 mA·h·g^−1^ at 0.2 A·g^−1^) and rate capability (30% vs. 8% capacity retained when the current density was increased from 0.2 A·g^−1^ to 5 A·g^−1^), as well as cycle life (44% vs. 17% capacity retention at 0.2 A·g^−1^ after 580 cycles). Overall, the present work provides a convenient route for obtaining MoS_2_ thin nanosheets for their advantageous use as anode material for lithium storage.

## 1. Introduction

In the last few decades, lithium-ion batteries (LIBs) have become a widespread energy storage solution for different purposes, mainly mobile applications that demand devices with high energy densities (e.g., portable electronic gadgets or electric vehicles [1]), but also stationary applications where cost considerations are probably a top priority over energy density (e.g., energy storage systems for the power grid [2]). To this day, graphite has been by far the anode material of choice for most commercial LIBs due to its appropriate features, such as laminar structure (allowing reversible accommodation of Li^+^ ions in substantial amounts), reduced volume expansion (~10%) and high electrical conductivity, as well as relatively low cost and wide availability [3]. However, a major drawback of graphite arises from its limited energy density, determined by its somewhat low gravimetric and volumetric theoretical capacity (372 mA·h·g^−1^ and 850 mA·h·cm^−3^, respectively) [3,4]. In concern of this drawback, several alternative materials have been studied with the aim of replacing graphite as a LIB anode [5]. Specifically, some members of the transition metal dichalcogenide (TMD) family of compounds are being considered promising candidates due to their layered structure, thus allowing the intercalation of ions in their lattice and opening the prospect for their implementation as LIB anodes [5,6]. More to the point, the wider availability and lower cost of MoS_2_ with respect to other TMDs, together with its outstanding gravimetric and volumetric theoretical capacity (670 mA·h·g^−1^ and 3390 mA·h·cm^−3^, respectively), justify the attraction of this TMD as a potential anode for LIBs [7]. In addition, given their layered nature, TMDs can be readily prepared as, or processed into, two-dimensional (2D) nanostructures, i.e., in the form of single-layer to several-layer nanosheets (NSs), which commonly demonstrate distinct properties when compared to their 3D, bulk counterparts [8,9]. Such a downsizing generally lends the resulting 2D materials a special interest for their use in energy storage systems, as it facilitates more extensive interactions with electrolyte and active redox species while shortening the pathway for the solid-state diffusion of charge carriers in the electrode [10]. In fact, previous reports in the literature suggest that 2D MoS_2_ could show a number of advantages as an anode for LIBs over other emerging materials (e.g., Si and Ge), including better cycling stability, higher rate performance and less influence of volumetric expansion [9]. In fact, recent reports where MoS_2_ was combined with materials with higher electrical conductivity highlight its potential as an electrode for lithium storage in LIBs [11,12,13,14], and in other types of lithium batteries, such as lithium–sulfur batteries [15].

As is normally the case when pursuing the practical implementation of any novel material, the real-life applications of 2D MoS_2_ NSs rely on the availability of the material in large amounts, preferentially obtained by cost-effective means. Currently, there are several bottom-up and top-down approaches to preparing MoS_2_ NSs. Among the bottom-up strategies, chemical vapor deposition and epitaxial growth are based on the reaction of sulfur and molybdenum precursors on an appropriate substrate, generally allowing precise control of the thickness, morphology and chemical doping of the resulting 2D crystals. However, these strategies are usually limited in their scalability beyond thin supported films, i.e., they cannot be used to access the large number of stand-alone NSs that would be desirable for their application as electrodes in LIBs and other electrochemical energy storage devices [16,17]. Likewise, solvothermal synthesis is a straightforward, scalable bottom-up approach to obtain 2D MoS_2_ at moderate temperatures, but the resulting NSs are in many cases obtained in an agglomerated form (e.g., nanoflowers), rather than as stand-alone objects, which may complicate their processing towards different applications [18]. On the other hand, top-down methods relying on the exfoliation of bulk MoS_2_ crystals appear to be particularly convenient for accessing stand-alone, processable 2D MoS_2_ flakes in substantial amounts [19,20]. For example, direct liquid-phase exfoliation of MoS_2_ in proper organic solvents [21] or water/surfactant solutions [22,23], prompted by ultrasound or shear forces, is a common strategy to attain stable colloidal dispersions of MoS_2_ NSs in considerable quantities. Nonetheless, this method typically suffers from low exfoliation yields and degrees (NSs comprising several layers rather than single/few layers), thus limiting their practical application potential.

Alternatively, the exfoliation of bulk MoS_2_ based on chemical or electrochemical intercalation with proper ionic species has also been a matter of considerable research interest over the last few years [24,25,26,27,28,29]. For instance, exfoliation by chemical and electrochemical intercalation of lithium is relatively widely employed to obtain substantial quantities of monolayer/few-layer MoS_2_ NSs, but the stringent conditions needed for their production (use of oxygen/water-free environments, long reaction times for chemical intercalation) decrease their practical attraction [27,30]. Moreover, (electro)chemical lithium intercalation generally induces a structural transition in the resulting exfoliated NSs, from the semiconducting 2H-phase to the metastable semimetallic 1T-phase, which is triggered by the massive injection of electrons in the host lattice associated with ion insertion [31,32]. Thus, achieving delamination into monolayer/few-layer NSs with no phase transition would strengthen the scope of applications where 2D MoS_2_ can display a prominent performance [20,33]. In this regard, the issue of phase transition upon ion intercalation has been circumvented by replacing the small Li^+^ cations with larger molecular species like tetraalkylammonium cations [34,35]. The larger size of the latter compared to Li^+^ limits the number of cations that can be packed into the interlayer spaces of MoS_2_, and consequently the number of electrons that are injected into the TMD lattice, thus preventing the 2H- to1T-phase transition associated with excess electrons [25]. However, the use of large cations does not generally lead to full exfoliation of the material, i.e., single-layer or even few-layer NSs are not usually the majority objects resulting from this exfoliation process [34]. Such an outcome has been rationalized by Lin et al. as resulting from a “self-retarding effect” associated with the insertion of large species [36]: as the intercalation of large cations proceeds in a given interlayer space, the mechanical strain induced in the TMD lattice progressively hinders subsequent intercalation into neighboring layers, preventing a complete exfoliation of MoS_2_ into single/few layers [36]. Exfoliation of bulk MoS_2_ can be also conducted by (electrochemical) intercalation of anions rather than cations [33]. Nonetheless, the intrinsically oxidizing conditions associated with the anodic process commonly imply a substantial transformation of the MoS_2_ lattice into MoO_x_ species, and thus a deterioration of many of the attractive features of 2D MoS_2_ [33].

Although substantial efforts have been devoted to this issue [33,34,35,36,37], attaining thinner 2H-phase MoS_2_ NSs by electrochemical delamination is still a challenge. To address this question, we hypothesize that choosing appropriate intercalating cations can ease the insertion process and lead to enhanced exfoliation degrees while retaining the original 2H-phase of the starting bulk MoS_2_ material. Specifically, while past work has focused on the use of tetraalkylammonium cations with alkyl substituents of identical length to drive MoS_2_ exfoliation [34,35,36,37], here, we propose their replacement by cations with alkyl substituents of dissimilar lengths, such as trimethylalkylammonium. The rationale behind this proposal is the following: an alkylammonium cation combining a (relatively) long alkyl chain with three very short chains would (1) still be large enough to prevent phase transformation in the resulting exfoliated MoS_2_ NSs and (2) have a small cross-sectional size in the direction of the long alkyl chain to promote a smoother intercalation compared to the case of larger, tetraalkylammonium cations with tetrahedral symmetry. Therefore, to probe this hypothesis, in this work, we have explored the use of a range of alkylammonium cations for the exfoliation of bulk MoS_2_ into thinner, 2H-phase NSs. The results indicate that trimethylalkylammonium ions with substituents of different lengths outperform their tetraalkylammonium counterparts for accessing few-layer MoS_2_ NSs. A discussion on the nature of this intercalation is addressed to account for such results. Additionally, the potential use of these new electrochemically derived 2H-phase MoS_2_ NSs as an electrode material for lithium storage is also investigated, delivering competitive results.

## 2. Experimental Section

### 2.1. Materials and Reagents

MoS_2_ natural crystals were purchased from SPI Supplies (West Chester, PA, USA), whereas MoS_2_ powder was provided by Alfa Aesar (Ward Hill, MA, USA). Platinum foil (25 × 25 × 0.025 mm^3^) was purchased from Goodfellow (Delson, QC, Canada). The following ammonium salts were supplied by Sigma-Aldrich (Saint Louis, MO, USA): tetramethylammonium chloride (TMACl); tetraethylammonium chloride (TEACl); tetrabutylammonium chloride (TBACl); tetrahexylammonium chloride (THACl); tetrahexylammonium tetrafluoroborate (THABF_4_); hexyltrimethylammonium chloride (HTMACl)/bromide (HTMABr); trimethyloctylammonium bromide (TMOABr); and hexadecyltrimethylammonium bromide, commonly referred to as cetrimonium bromide or cetyltrimethylbromide (CTAB). As for the solvents, propylene carbonate (PC), N,N-dimethylformamide (DMF) and N-methylpirrolidone (NMP) were obtained from Sigma-Aldrich, whereas acetone was purchased from VWR (Radnor, PA, USA). Multiwall carbon nanotubes and carbon black Super C65 were purchased from Arkema (Colombes, France) and Timcal (Bodio, Switzerland), respectively. Polyvinylidene difluoride (PVDF, Solef) and glass fiber paper (thickness 670 µm) were acquired from Solvay (Brussels, Belgium) and Whatman^®^ (Little Chalfont, UK), respectively. Li foil was purchased from Sigma-Aldrich. A 1 M lithium hexafluorophosphate solution in ethylene carbonate/dimethyl carbonate (1/1 weight ratio) (LP30) was supplied by Solvionic (Toulouse, France). Coin cells CR2032 were provided by S4R (Anaheim, CA, USA). Milli-Q deionized water (Millipore Corporation, Billerica, MA, USA; resistivity: 18.2 MΩ cm) was used for all the experiments.

### 2.2. Cathodic Exfoliation of MoS_2_ Crystals

First of all, a ~4 × 6 × 1 mm^3^ piece of MoS_2_ was cut from the as-received crystal and cleaved on both sides to expose a fresh surface by removing the top layers. The electrochemical exfoliation was carried out in a two-electrode setup under cathodic conditions, using the piece of MoS_2_ crystal and platinum foil as the working and counter electrodes, respectively, and an organic solution of a given quaternary ammonium salt as the electrolytic medium (15 mL). Both electrodes were connected to a DC power supply (E3633A apparatus, from Keysight Technologies, Santa Monica, CA, USA and Agilent 6614C power supply from Agilent, Santa Clara, CA, USA were indistinctly used) with crocodile clips and kept at a distance of ~2 cm from each other in the electrolytic solution. In this configuration, about two-thirds of the MoS_2_ electrode was immersed in the electrolyte. A negative voltage (−8 V) was then applied to the MoS_2_ electrode for 30 min, during which it was generally seen to swell and fan out starting from its free (non-clipped) end. Also, some of the outer layers of the crystal eventually detached from the electrode surface. After the cathodic treatment, the MoS_2_ electrode was extracted from the solution and the lower part of the crystal was separated from the part which had not been submerged with a scalpel. The portions detached from the electrode during the cathodic treatment were also gathered with a spatula from the bottom of the electrolytic medium vessel and stored in DMF with the rest of the electrochemically treated MoS_2_ for further use. It was preferred not to dry the product to prevent its expansion from reversing by deintercalation.

To obtain individualized MoS_2_ NSs from the electrochemically treated material, the latter was transferred to DMF at a nominal concentration of 1 mg mL^−1^ and bath-sonicated (Ultrasons systems from J.P. Selecta, Cerdanyola del Vallès, Spain, 40 kHz) during 5 h (changing from one bath to another to avoid the overheating of water). The obtained suspensions were washed to eliminate any remnants of the electrolytic medium through four consecutive sedimentation/re-suspension cycles. In each cycle, the dispersions were first subjected to centrifugation (Eppendorf 5430 microcentrifuge from Eppendorf, Hamburg, Germany) at 10,000× *g* for 25 min to sediment all the material, followed by replacement of 75% of the supernatant by an equal volume of DMF, and finally redispersion by bath sonication (~3 min) before the next washing sequence. After the four washing cycles, the dispersions were left to rest undisturbed overnight to allow for the sedimentation of non- and poorly exfoliated fractions. A ~75% of the supernatant volume, expected to contain the well electrochemically exfoliated MoS_2_ NSs, hereinafter referred to as ee-MoS_2_, was collected for further use.

For comparison purposes, liquid-phase exfoliated MoS_2_ (lpe-MoS_2_) was also prepared from bulk MoS_2_ powder as previously reported [38]. Briefly, the protocol was the same as described above for the extraction of ee-MoS_2_ NSs but omitting the washing step and starting from a nominal concentration of 30 mg mL^−1^ of bulk MoS_2_ powder.

### 2.3. Characterization Techniques

The materials were characterized by UV-Vis absorption spectroscopy, field-emission scanning electron microscopy (FE-SEM), high-resolution transmission electron microscopy (HR-TEM), energy dispersive X-ray spectroscopy (EDX), selected area diffraction (SAED), X-ray diffraction (XRD), atomic force microscopy (AFM), X-ray photoelectron spectroscopy (XPS), Raman spectroscopy, krypton physisorption, as well as dynamic light scattering (DLS). UV-Vis absorption spectra were recorded on a double-beam Genesys 180 spectrophotometer (Thermo Fischer Scientific, Waltham, MA, USA) with a wavelength step of 0.5 nm. FE-SEM imaging was performed on a Quanta FEG instrument (FEI Company, Hillsboro, OR, USA) that worked at a bias voltage of 20–25 kV. HR-TEM and SAED were carried out with a JEOL JEM-2100F instrument (JEOL, Tokyo, Japan) operated at an accelerating voltage of 200 kV and equipped with a 14-bit Gatan Orius SC600 CCD camera (Gatan, CA, USA), and bright-field and high-angle annular dark field detectors (JEOL). To prepare graphene specimens for HR-TEM, a few microliters of their aqueous suspensions were dropped onto copper grids (200 square mesh) covered with a lacey carbon film (Micro to Nano Innovative Microscopy Supplies, Haarlem, The Netherlands) and then allowed to dry under ambient conditions. XRD diffractograms were acquired on a Bruker D8 Advance diffractometer (Bruker, Karlsruhe, Germany) equipped with a Cu K_α_ anode. The data were recorded in a 2θ range of 5°–80° with a step size of 0.015° and time per step of 6 s for XRD measurements, the starting TMD powders were used as received, while the exfoliated materials were prepared in the form of films by filtering from dispersion through polycarbonate membranes with a pore size of 50 μm (Nucleopore™, Whatman^®^). AFM images were recorded with a Veeco Nanoscope-IIIa (Digital Instruments, Tonawanda, NY, USA) working in the tapping mode of operation. For AFM imaging, a few microliters of polyvinylpyrrolidone-assisted MoS_2_ dispersion were drop-cast onto atomically flat Si/SiO_2_ (2000 nm SiO_2_) substrates. XPS was measured on a spectrometer equipped with a monochromatic Al K_α_ X-ray source (14.00 KV, 175 W) and a Phoibos 100 hemispherical electron energy analyzer (SPECS Surface Nano Analysis, Berlin, Germany) and working at a pressure below 10^−7^ Pa. The XPS spectra were recorded at a take-off angle of 90°, analyzing the photoexcited electrons in the constant pass energy mode at a pass energy of 50 and 10 eV for survey and high-resolution core-level spectra, respectively. Raman spectra were registered with a Renishaw in Via Qontor apparatus (Renishaw, London, UK), working at a laser excitation wavelength of 532 nm (green line) and using an incident laser power sufficiently low (<0.5 mW) so as to minimize sample damage. Graphene samples for XPS and Raman spectroscopy were prepared by first pre-concentrating their dispersions in DMF, then transferring to a small volume of water and finally sequentially drop-casting small volumes of dispersion onto circular stainless steel discs (12 mm in diameter) and allowing them to dry at ambient conditions until a continuous film was seen to uniformly cover the whole disc. Due to the low values of the specific surface area expected for the parent MoS_2_ materials, mostly in the case of the MoS_2_ crystals, krypton rather than more common nitrogen physisorption was used for its determination, given that the former is more suitable for determining surface areas below 5 m^2^·g^−1^ [39]. Krypton physisorption at 77 K was measured in an ASAP 2420 surface area and porosity analyzer (Micromeritics, Norcross, GA, USA). Prior to the adsorption experiments, the samples were degassed under a vacuum at 120 °C for 12 h. The specific surface areas were obtained from the adsorption branch of the Kr isotherms by the standard Brunauer–Emmett–Teller (BET) method in the relative pressure range from 0.04 to 0.24. DLS measurements of MoS_2_ dispersions in DMF were carried out on a Litesizer DLS 500 instrument (Anton Paar, Graz, Austria), equipped with a 658 nm wavelength laser. Ten replicates of each sample were made at a backscatter (angle of 175°).

### 2.4. Electrochemical Measurements and Post-Mortem Characterization of the Electrodes

Both ee-MoS_2_ and lpe-MoS_2_ were tested as active materials for electrodes for Li storage in coin cells in a half-cell configuration, performing as an anode for LIB. The exfoliated MoS_2_ materials were subjected to lyophilization prior to their use. First, lpe-MoS_2_ or ee-MoS_2_ NSs colloidal dispersions in DMF were subjected to high-speed centrifugation until complete sedimentation of the corresponding material was achieved. Then, 75% of the supernatant was discarded and the sedimented MoS_2_ powders were redispersed in the remaining volume in order to increase the concentration of the resulting dispersions. After several concentration steps, the sedimented powders were subjected to three washing cycles (as previously described in Section 2.3), and then freeze-dried (Telstar Lyoquest-85 from Telstar, Terrassa, Spain) for 4 days. The working electrode consisted of a mixture of the electroactive MoS_2_ material with multi-wall carbon nanotubes and carbon black as the conductive additives, and PVDF as the binder in a weight ratio of 54:16:20:10. To prepare such a mixture, appropriate masses of the first three components were added to the corresponding weighted volume of 5% PVDF/NMP. In order to produce a slurry, some pure NMP was added to the mixture which was then subjected to high-shear mixing (Ultra-Turrax T25 from IKA, Staufen im Breisgau, Germany) for 20 min. The resulting homogeneous slurry was cast onto chemically modified Cu foil and evenly distributed on its surface using a Doctor Blade. The wet covered substrate was subjected to slow drying for several hours at low temperature (~40–50 °C) on a heating plate, followed by a second drying step in a vacuum oven at 80 °C for 12 h. The coated Cu foil was cut into 10 mm diameter discs with a total mass loading between 1.4 and 2.5 mg·cm^−2^. A piece of Li foil was used as both the counter and reference electrode in a half-cell configuration. The coin cells were assembled in a glove box (Jacomex, Dagenux, France) under an argon atmosphere, with LP30 as the electrolyte and two stacked glass fiber filters as the separator. Prior to that, the different components (electrodes, separator, etc.) had been dried in an oven (B-545 from Büchi, Flawil, Switzerland) at 120 °C for 12 h.

The electrochemical response of the materials was monitored with a VMP3 potentiostat (Biologic, Grenoble, France), recording open circuit voltage (OCV), cyclic voltammograms (CVs) at different scan rates, electrochemical impedance spectroscopy (EIS) and galvanostatic charge–discharge (GCD) profiles with potential limitation at different current densities. Prior to any electrochemical measurement, the coin cells were left unbiased under OCV for 4 h to ensure wetting with the electrolyte.

Post-mortem microscopic studies of the electrodes after long-term cycling were conducted by FE-SEM. The used coin cells were disassembled using a hydraulic crimping machine. The electrodes were washed six times through several hours-long immersions in dimethyl carbonate, which was the same solvent of the electrolyte used in the electrochemical measurements (LP30). After that, most of the solvent was removed with the help of a pipette, and the electrode was dried first under ambient conditions and then overnight under a vacuum at room temperature. If present, the dry remains of the glass fiber separator were removed with tweezers, taking care not to scratch the surface of the electrode.

## 3. Results and Discussion

### 3.1. Optimization of the Cathodic Exfoliation Protocol

**Figure 1:**
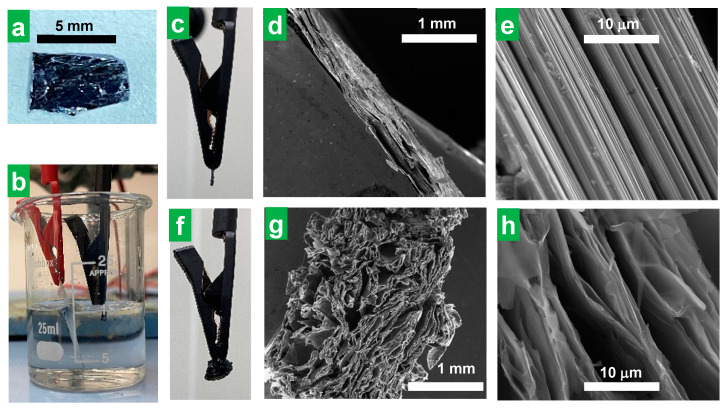
Cathodic expansion of bulk MoS_2_ crystals. Digital photographs of (**a**) a typical bulk MoS_2_ crystal used as a cathode for electrochemical exfoliation and (**b**) the experimental set-up for the cathodic exfoliation of MoS_2_ using quaternary ammonium salt solutions in polycarbonate as the electrolyte. (**c**) Digital photograph and (**d**,**e**) FE-SEM micrographs at different magnifications of the edge of the MoS_2_ cathode before electrochemical expansion. (**f**) Digital photograph and (**g**,**h**) SEM micrographs of the MoS_2_ cathode after electrochemical expansion at −8 V for 30 min using 0.05 M HTMABr in PC as the electrolytic medium.

In the search for an electrochemical exfoliation protocol for the preparation of thin MoS_2_ NSs, cations with alkyl substituents of dissimilar lengths, specifically, alkyltrimethylammonium salts, have been tested as electrolytes, in contrast with previous studies, where tetraalkylammonium cations with alkyl substituents of equal length were explored for such use [34,35,36,37]. To test our hypothesis that alkyltrimethylammonium cations (with trigonal pyramidal symmetry) could be more effective as electrolytes than tetraalkyllammonium cations (tetrahedral symmetry) in accessing thinner exfoliated MoS_2_ flakes through cathodic delamination, the performance of both types of compounds was compared on an equal footing. In a typical experiment, cathodic exfoliation was carried out in a two-electrode configuration, using MoS_2_ pieces (~4 × 6 × 1 mm^3^ in size, Figure 1a) as the working electrode, platinum foil as the counter electrode, and a solution of a certain ammonium salt in polycarbonate (PC) as the electrolytic medium (Figure 1b, see the Section 2 for further details). In an optimized procedure (−8 V, 0.05 M HTMABr in PC), upon application of the negative bias voltage to the MoS_2_ cathode (Figure 1c), an accordion-like swelling of the former became visible to the naked eye (Figure 1f, Supplementary Movie S1). After completion of the cathodic expansion step, the electrochemically treated part of the MoS_2_ crystal was separated from the non-treated portion (see the Section 2 for details) and characterized. The overall field-emission scanning electron microscopy (FE-SEM) image of the expanded portion of the cathode (Figure 1g), consisting of separated lamellae, contrasted sharply with the compact nature observed for the starting crystal at the same magnification (Figure 1d). A more detailed inspection confirmed that the lamellae were made up of thin, wrinkled layers separated by micrometer or sub-micrometer-sized voids (Figure 1h), again in contrast with the morphology of the starting, untreated MoS_2_ piece, which was dominated by non-expanded and closed-packed layers (Figure 1e). Simultaneous to the expansion, gas bubble evolution in the cathode and the generation of a yellow substance in the counter electrode were observed when HTMABr was used as the electrolyte (see Supplementary Movie S1). The nature and production mechanism of the released substances will be discussed below. Nevertheless, all the ammonium salts tested as electrolytes but the smallest, TMACl, led to some expansion of the MoS_2_ crystal whether there was gas evolution and/or yellow substance generation or not.

**Figure 2:**
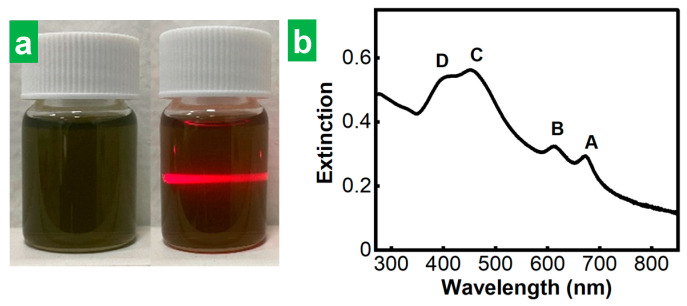
Electrochemically exfoliated MoS_2_ colloidal dispersions. (**a**) Digital photographs of MoS_2_ colloidal dispersions in DMF obtained after ultrasound-assisted exfoliation (**left**) showing the Tyndall effect (**right**). (**b**) UV-Vis extinction spectrum of the dispersion in (**a**). The excitonic peaks A–D, characteristic of the 2H-MoS_2_ phase are labeled for clarity.

To extract individual, stand-alone MoS_2_ NSs from the cathodically expanded materials, they were transferred to DMF at a nominal concentration of 1 mg mL^−1^, and subjected first to sonication for 5 h and then to 4 washing cycles to remove any remaining electrolytic medium from the cathodic step (see Section 2 for details). The dispersions obtained after discarding the non- and poorly exfoliated fractions (see Section 2 for details) exhibited the Tyndall effect, which is indicative of the presence of a colloid (Figure 2a, right). They also displayed the characteristic green tone of nanostructured MoS_2_ in the thermodynamically stable 2H-phase (Figure 2a, left) [8,40]. Indeed, their UV-Vis extinction spectrum (Figure 2b) featured the excitonic bands (A, B, C and D) characteristic of semiconducting 2H-phase MoS_2_ [8]. Although the data shown in Figure 2 correspond to the dispersions obtained with HTMABr as an electrolyte, all the electrolytes that allowed MoS_2_ crystal expansion (i.e., all the ammonium salts tested here except TMACl) yielded greenish MoS_2_ dispersions which showed UV-Vis spectra typical of 2H-phase MoS_2_. This provided confirmation that the phase transformation from the stable 2H phase of the starting natural crystal to the metastable 1T-phase in the resulting exfoliated MoS_2_ NSs was prevented for every electrolyte. This means that all the electrolytes were large enough to limit the number of molecules that can fit into the interlayer species of the host crystal and thus the number of electrons injected per MoS_2_ formula unit to values below the phase-transition threshold [41]. The preservation of the 2H-phase of the starting bulk MoS_2_ material in the final exfoliated material obtained upon ammonium cations intercalation had already been shown in previous reports for the case of some of the larger cross-section, tetraalkylammonium cations with alkyl substituents of identical length tested here [34,35,36,37]. However, it was yet to be confirmed for the alkyltrimethylammonium cations with a smaller cross-sectional size, which were the subject of the current study.

To test our second hypothesis that alkyltrimethylammonium cations, with a smaller cross-section in the direction of the long alkyl chain, could yield thinner MoS_2_ flakes compared to the case of tetraalkylammonium cations, the thickness of MoS_2_ NSs obtained with the different electrolytes were estimated and compared. Based on the metrics previously developed by Backes et al. for 2H-MoS_2_ NSs, where the wavelength of the maximum of the A excitonic band of dispersion was shown to correlate with the thickness of the flakes therein [8], the position of A excitonic band was taken as a proxy of the thickness of the MoS_2_ NSs. When the electrolytes were compared under equal conditions (bias voltage of −8 V, electrolyte concentration of 0.05 M in PC) for the cathodic expansion of MoS_2_, HTMABr led to the lowest wavelength for the A band of the corresponding MoS_2_ dispersion amongst all the tested electrolytes, as can be seen from the data gathered in Table 1. Therefore, HTMABr was selected as the optimal electrolyte because it allowed dispersions with the thinnest flakes.

### 3.2. Physicochemical Characterization of the Cathodically Exfoliated Products

**Figure 3:**
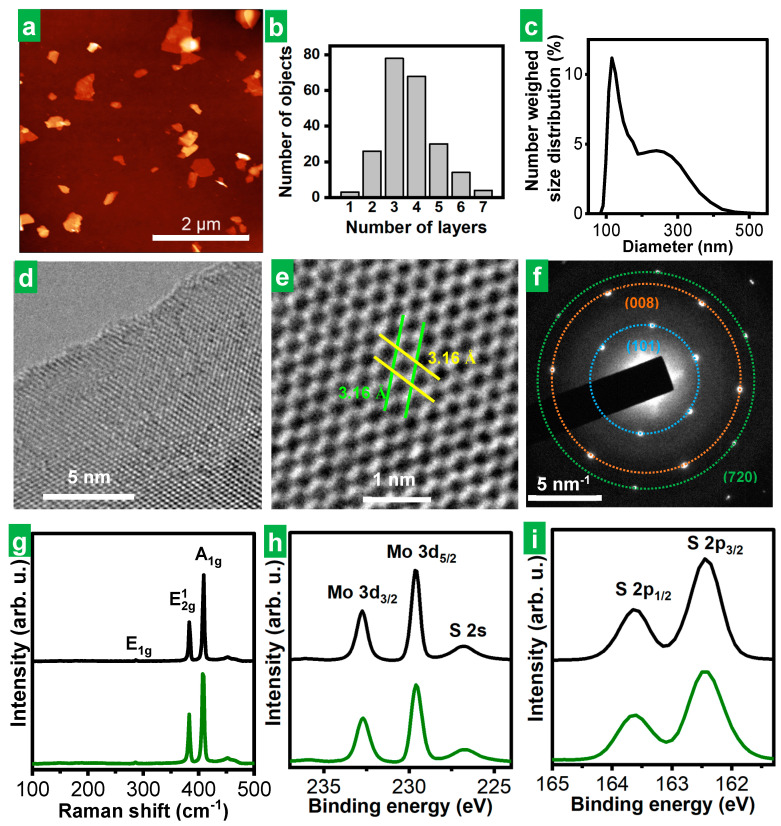
Microscopic and spectroscopic characterization of electrochemically exfoliated MoS_2_ colloidal dispersions. (**a**) Representative AFM image of the MoS_2_ nanosheets deposited onto a Si/SiO_2_ substrate from dispersion. (**b**) Histogram of the apparent thickness of MoS_2_ nanosheets derived from a pool of over 200 nanosheets measured from the AFM images. (**c**) DLS-derived number-weighed hydrodynamic diameter distribution for MoS_2_ dispersion in DMF. (**d**,**e**) Representative HR-TEM images of the MoS_2_ basal planes at different magnifications. The parallel lines in (**e**) assist in visualizing *a* and *b* cell parameters in the hexagonal cell of the MoS_2_ lattice. (**f**) SAED pattern of the MoS_2_ lattice with an indication of the families of planes involved in the observed diffractions. (**g**) Typical Raman spectra of the starting bulk MoS_2_ bulk crystal (black trace) and cathodically exfoliated MoS_2_ (green trace). (**h**,**i**) Typical XPS spectra of (**h**) Mo 3d and (**i**) S 2p core levels for bulk MoS_2_ crystal (black trace) and cathodically exfoliated (green trace) MoS_2_ NSs. The main bands have been labeled for clarity.

Microscopic and spectroscopic characterization of the solvent-extracted material derived from the cathodically expanded MoS_2_ using HTMABr as the electrolyte confirmed that it consisted of individual, stand-alone, multilayer 2H-phase MoS_2_ NSs of high structural quality. Indeed, the atomic force microscopy (AFM) images of the product depicted NSs of irregular polygonal shapes (Figure 3a) with lateral sizes typically in the range of several hundreds of nanometers. Assuming a thickness of MoS_2_ monolayer of ~1.9 nm and a height offset of ~1 nm [8], we concluded that the exfoliated NSs were typically between 2 and 6 monolayers thick (Figure 3b). The electrochemically exfoliated MoS_2_ material obtained in this work compared favorably in terms of flake thinness with a typical liquid-phase exfoliated MoS_2_ material, lpe-MoS_2_ (see the Section 2 for details on its preparation), which displayed thickness between ~7 and 30 monolayers (see Figure S1b in Section S1 of the Supplementary Materials). As previously indicated by the A-band position for MoS_2_ dispersions (Table 1), ee-MoS_2_ nanosheets obtained using HTMABr as electrolyte were also found to be thinner on average than those obtained different ammonium salts as electrolytes by AFM (see representative images and the corresponding histograms in Figure S2, Section S1 of the Supplementary Materials, for the case MoS_2_ obtained with THABF_4_ and TMOABr). Dynamic light scattering (DLS) measurements yielded hydrodynamic diameters for the colloidally dispersed ee-MoS_2_ NSs roughly between 80 and 460 nm (Figure 3c). Using the quantitative relationship between hydrodynamic diameter and NS lateral size previously developed for graphene and other 2D materials [42], the actual lateral size of the MoS_2_ NSs was estimated to stretch between 50 and 700 nm, which was in reasonable agreement with the microscopy results (see Figure 3a). Figure S2 HR-TEM images of the MoS_2_ revealed their high structural quality (Figure 3d). Atomic resolution images of the basal planes and the corresponding SAED patterns (Figure 3e and Figure 3f, respectively) disclosed their hexagonal symmetry and unit cell parameters.

Raman spectroscopy further confirmed that the thermodynamically stable 2H-phase of the starting bulk MoS_2_ natural crystal (Figure 3g, black trace) was preserved in the exfoliated NSs (Figure 3g, green trace), as both materials showed equivalent spectra featuring the phonon bands expected for such structure [43]. This was only to be expected from the fact that the parent dispersion of the film drop-cast for Raman analysis yielded UV-Vis spectra typical of 2H-phase MoS_2_ (see Figure 2b). XRD analysis provided diffractograms typical of 2H-MoS_2_ (Figure S3a,c in Section S1 of the Supplementary Materials) as well. XPS spectroscopy showed that the chemical nature of the material was not altered either to any significant extent during its exfoliation and subsequent processing. Indeed, the core-level spectra of the main elements, molybdenum and sulfur, of the exfoliated material (Figure 3h and Figure 3i, respectively, green trace) were essentially identical to those of the starting bulk MoS_2_ crystal (Figure 3h,i, black trace). Certainly, the high resolution, core-level Mo 3d, S 2s and S2p spectra (Figure 3h,i) exhibited the binding energies (BE) expected for such elements in 2H-phase MoS_2_, namely, ~229.6 eV for Mo 3d_5/2_ and 232.8 eV for Mo 3d_3/2_ for Mo^4+^ with trigonal prismatic coordination to six sulfide ions, and ~226.8 eV for S 2s, ~162.4 eV for S 2p_3/2_ and ~163.6 eV for S 2p_1/2_ for S^2−^ with pyramidal coordination to three Mo^4+^ [44]. Apart from that, no oxidized Mo or S species could be found at the level of detection of XPS (i.e., above a few tenths of at%), either in the starting material or in its exfoliated counterpart.

By way of comparison, Figures S1 and S3d in Section S1 of the Supplementary Materials show the results of the characterization, carried out in a completely analogous way to that of ee-MoS_2_, with MoS_2_ obtained by liquid-phase ultrasound-assisted exfoliation of bulk MoS_2_ powder. As in the case of the electrochemically delaminated material obtained from bulk MoS_2_ crystals, XRD results as well as microscopic and spectroscopic characterization confirmed that the 2H-phase and the chemical nature of the starting bulk MoS_2_ powder were preserved in the material derived from it by liquid-phase exfoliation (lpe-MoS_2_). As for the dimensions of the exfoliated NSs, their lateral size, shown by AFM and estimated from DLS hydrodynamic diameters (Figure S1a,c in the Supplementary Materials) ranged between 40 and 425 nm, below that of ee-MoS_2_. However, as said above, the ee-MoS_2_ flakes were much thinner than their lpe-MoS_2_ counterparts (compare Figure 3b and Figure S1b), which is expected to lead to increased surface area. As will be explained later, thinner ee-MoS_2_ NSs were found to perform better than thicker lpe-MoS_2_ in their application to electrochemical energy storage, where high accessibility of the electrolyte to the surface is crucial. However, the specific surface areas of the ee-MoS_2_ and lpe-MoS_2_ materials measured by gas physisorption were found to be similar to each other, both being in the range of several tens of m^2^·g^−1^, which is in the order of the values reported in the literature for nanostructured MoS_2_ materials [7,13]. Although the specific surface areas of the exfoliated materials were not significantly different from each other, they were notably higher, as expected, than those of the parent bulk materials, which showed values in the units of m^2^·g^−1^ range for the powder and in the hundredths of m^2^·g^−1^ range in the case of the crystal (just the geometrical surface). Nevertheless, these surface areas, measured by gas adsorption on dry powders, need not be representative of the area exposed to the electrolyte in their application to electrochemical energy storage. In fact, the dry powder, even if obtained by freeze-drying, must consist of re-stacked 2D films. Although it is not known how exactly this re-stacking occurs or how it depends on the thickness of the sheets, the fact that thicker sheets tend to be stiffer makes it reasonable to think that they will re-stack worse than the thinner ones and, therefore, tend to lose less area with respect to the case without re-stacking. Indeed, there are previous instances in the literature where a thinner, electrochemically exfoliated 2D material (e.g., graphene) is shown to lead to more compact, less porous re-stacked films than its thicker, liquid-phase exfoliated counterpart [45].

### 3.3. Rationalization of the Cathodic Exfoliation Process

Having established that the alkyltrimethylammonium cations, with a smaller cross-section in the direction of the long alkyl chain, yield thinner MoS_2_ flakes compared to the case of larger tetraalkylammonium cations, it is pertinent to discuss the factors that lead to such different performance. Let us first analyze the nature of the intercalation process. Driven by the negative electrochemical potential, a certain number *x* of ammonium cations (R_3_R’N^+^, where R and R’ are alkyl radicals; R = R’ for tetraalkylammonium; R = CH_3_ ≠ R’ for trimethylalkylammonium) intercalates between the MoS_2_ cathode layers in the electrochemical cell, generating an intercalation compound [Equation (1)]:

MoS_2_ + *x*R_3_R’N^+^ + *x*e^−^ → (R_3_R’N^+^)_x_MoS_2_^x−^ (1)

According to previous theoretical studies, the phase transition from the semiconductor 2H-phase to the semimetal 1T-phase in TMDs occurs only when the electron injection is above a certain threshold, which in the case of MoS_2_ acquires the relatively high value of 0.29–0.35 electrons per formula unit [41]. The absence of phase transition in our MoS_2_ exfoliated materials revealed by their characterization (see previous section) demonstrates that this threshold was not exceeded by intercalation with the tested substituted ammonium species. Subsequently, the decomposition of the cations [Equation (2)] can contribute to enlarging the inter-layer distance of MoS_2_, favoring further intercalation and weakening the van der Waals interactions, eventually pushing the layers apart. In fact, tetraalkylammonium can accept an electron and reduce to trialkylamine and an alkyl radical, which can react with another alkyl radical to yield an alkene [46].

(R_3_R’N^+^)_x_MoS_2_^x−^ → MoS_2_ + trialkylamines + alkenes (2)

In this respect, cathodic gas generation was clearly observed for every tested electrolyte (see, for example, Supplementary Movies S1 and S2 for HTMABr and TEACl, respectively) but for TBACl and THACl (see Supplementary Movie S3 for THACl). The generation of cathodic gas depends on whether or not the given alkyl-substituted ammonium has the potential to give rise to low-molecular-weight alkenes by electrochemical reduction. In the case of TBA^+^ and THA^+^, the alkyl substituents are too long to generate gaseous alkenes by reduction, producing liquids instead.

**Figure 4:**
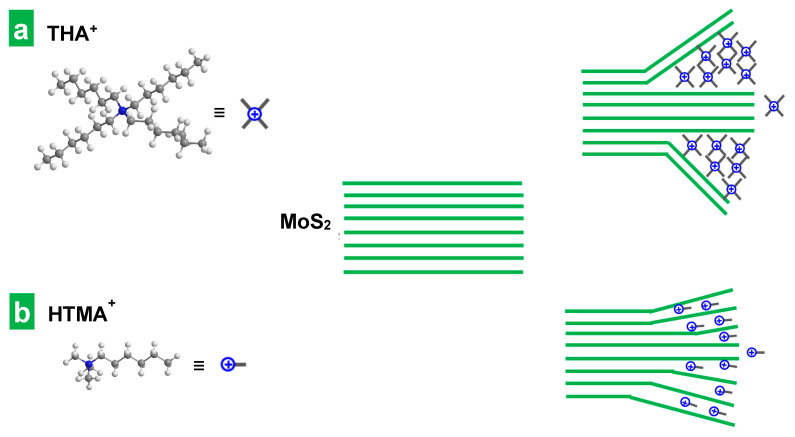
Schematic of the intercalation and exfoliation of layered MoS_2_ by tetraalkylammonium and alkyltrimethylammonium cations. Intercalation and exfoliation of MoS_2_ with (**a**) larger cross-section tetraalkylammonium cations, and (**b**) alkyltrimethylammonium cations, with a smaller cross-section in the direction of the long alkyl chain.

Obviously, the size of the cations (or rather their effective intercalation cross-section) is a crucial factor in the intercalation process. Indeed, as explained above, for sufficiently large cations, the intercalation promoted a volume expansion of the MoS_2_ crystal that facilitated its subsequent ultrasound-assisted exfoliation (see Table 1). Indeed, for tetraalkylammonium electrolytes with identical substituents, the intensity of the electrochemical expansion phenomenon was observed to increase with the length of the alkyl chain, i.e., with the size of the intercalated cation. In fact, the smallest cation, TMA^+^, led to no expansion at all of the MoS_2_ cathode, and thus the subsequent extraction of MoS_2_ NSs by sonication was not possible (see Table 1). The ineffectiveness of this intercalant to expand the host crystal can be traced back to its diameter (0.56 nm) [46] being smaller than the interlayer distance of the crystal (0.61 nm) [34]. The next tetraalkylammonium cation in size, TEA^+^ (0.67 nm) [46], did give rise to some expansion (see Supplementary Movie S2), but the subsequent ultrasound-assisted exfoliation of the portion subjected to electrochemical treatment led to very slightly green-colored, diluted dispersions, which was indicative of incomplete, scarce exfoliation. For the larger, butyl- and hexyl-substituted tetraalkylammonium cations, the increase in the volume of the MoS_2_ crystals upon electrochemical treatment was much more pronounced (see Supplementary Movie S3 for the electrochemical expansion using THA^+^) and led to significant exfoliation during the subsequent ultrasonication step. Thus, the extent of the electrochemical expansion was seen to determine the amount of exfoliated material extracted in the subsequent bath sonication step, which was non-existent for the smallest TMA^+^, negligible for TEA^+^, but increasingly significant for the larger cations TBA^+^ and THA^+^. Although, in principle, the more vigorous electrochemical expansion process observed for TBA^+^ and THA^+^ seemed advantageous, such intercalants were still not optimal. In fact, they led to an early saturation of the intercalation process, which did not allow for the expansion of the whole MoS_2_ piece to take place over the 30 min electrochemical treatment. Indeed, the cathode swelling was very fast for the first 10 min, but it became arrested afterward (see Supplementary Movie S3), consistently leaving a preserved, internal portion of the MoS_2_ piece with no appreciable expansion to the naked eye. As mentioned before, this type of phenomenon—the incomplete intercalation of bulk TMD crystals by large tetraalkylammonium cations—has been previously reported and explained by Lin at al. on the basis of a so-called “self-retarding effect” [36]. According to this explanation, during the first stages of the electrochemical treatment, the intercalation takes place mainly within the interlayer gaps near the TMD crystal surface, promoting a great initial expansion in its vicinity [36]. Then, the presence of the already intercalated bulky ammonium cations hinders the advance of the process in the neighboring layers due to the appearance of steric and electrostatic repulsions. The presence of accumulated mechanical strain at the interface between expanded and pristine areas in the host crystal prevents its complete, uniform intercalation, yielding thicker, multilayer NSs (Figure 4a). The trend of increasing thickness observed in this work for the series of exfoliated materials obtained with increasingly larger tetraalkylammonium cations (see Table 1) agrees with this explanation.

However, when alkyltrimethylammonium cations, with a smaller cross-section in the direction of the long alkyl chain, were used as electrolytes, the intercalation they promoted was found to be smoother. As the long alkyl chains of the three tested alkyltrimethylammonium cations are in fact equal (HTMA^+^ vs THA^+^) or longer (TMOA^+^, CTA^+^) than the substituents in the four tetraalkylammonium, the smoother intercalation of the former suggests that their long chains are oriented in parallel instead of in perpendicular to the atomic layers. This is supported by a previous study on an electrochemical molecular intercalation approach to obtain 2D superlattices where the intercalation of trimethylalkylammonium cations with alkyl substituents of dissimilar lengths was found to produce MoS_2_ superlattices with very similar interlayer expansion [47], indicating that the species preferentially intercalate through their smaller, common trimethylammonium “head”. Indeed, using HTMA^+^, unlike the case of its fully hexyl-substituted counterpart THA^+^, the expansion of the electrode was seen to proceed at a steadier pace along the whole electrochemical treatment (compare Supplementary Movies S1 and S3) leaving no unexpanded internal regions behind. Furthermore, the thickness of the MoS_2_ flakes obtained with HTMA^+^ was smaller, as indicated by the lower position of the A exciton of the corresponding dispersions (see Table 1). All these facts suggested that the self-retarding effect was alleviated in these conditions. As seen in Supplementary Movie S1, the smaller size and cross-section of HTMA^+^ decreased the rate and extent of the initial volume expansion of the MoS_2_ piece, which must have lowered the steric repulsion and the mechanical strain therein and facilitated further delamination (see Figure 4b). As previously found in the case of the tetraalkylammonium series, the thickness of the resulting MoS_2_ NSs increased with the size of the electrolyte. Indeed, as the length of the dissimilar alkyl chain grew along the series of homologous alkyltrimethylammonium cations, the wavelength of the A exciton increased (see Table 1). This must come from the increase in the steric repulsion and mechanical strain induced by the intercalation of bulkier species. HTMABr was thus selected as the best among the tested electrolytes and its concentration was varied in the range from 0.01 M to 0.1 M in search of an optimum. A concentration of 0.05 M was found to be optimal: while lower concentrations led to less vigorous expansion of the MoS_2_ piece, higher concentrations promoted an early arrest of the swelling. The latter was presumably due to the saturation of the intercalation process by the presence of an excessive amount of intercalated electrolyte in near-surface interlayer spaces of the crystal.

On the other hand, the corresponding anions of the electrolytic salts oxidize in the anode. For instance, the intense yellow substance released from the anode when bromide salts (HTMABr, TMOABr and CTAB) were used (see Supplementary Movie S1 for the case of HTMABr) was molecular bromine, which was generated by the oxidation of the bromide counterions. This was verified by substitution of the bromide counterion by chlorine, using HTMACl. In the latter case, the pale greenish-yellow color of molecular chlorine was observed instead of the intense yellow color of bromine.

### 3.4. Lithium Storage Performance of the Cathodically Delaminated MoS_2_ Material

**Figure 5:**
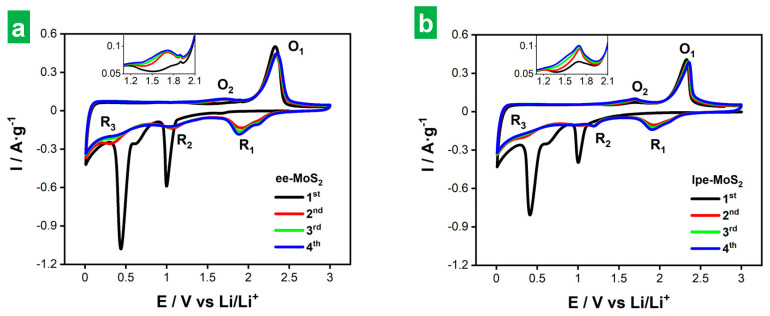
First four cyclic voltammograms (CVs) of electrodes based on exfoliated MoS_2_ materials. CVs of the first four cycles at 0.2 mV s^−1^ of (**a**) ee-MoS_2_ and (**b**) lpe-MoS_2_ electrodes. The insets show a magnification in the voltage range of 0.8–1.9 V.

The lower thickness of cathodically delaminated MoS_2_ NSs is expected to be beneficial for its use in electrochemical charge storage applications. In order to verify this assumption, the Li storage performance of both the thinner cathodically delaminated MoS_2_ (ee-MoS_2_) and the thicker liquid-phase exfoliated material (lpe-MoS_2_) was evaluated under the same conditions for comparison. The tests were carried out in a half-cell configuration, using delaminated MoS_2_ as the working electrode in combination with conductive additives (a mixture of carbon nanotubes and carbon black) and a binder. In the case of carbon nanotubes, their role was to improve not only the electrical conductivity of the electrode but also the mechanical properties, dampening the volumetric changes during charge/discharge cycles [48]. A weight ratio of 54:16:20:10 was used for exfoliated MoS_2_/carbon nanotubes/carbon black/binder. The low relative content in active material ensures the absence of kinetic restrictions. The latter is desirable to assess the potential of the material for Li storage, although the formulation would have to be optimized for its actual implementation. However, we note that the mass loading of the active materials was ~0.7–1.3 g cm^−2^, which is within the usual range used for tests in half-cell configuration. The morphology of the electrodes was revealed by FE-SEM (Figure S5 in Section S3 of the Supplementary Materials), showing a homogeneous appearance on a micrometer scale (Figure S5a,b) and the expected features from the individual nanostructured components of the mixture at the nanometer scale (Figure S5c,d). Specifically, nanosheets with dimensions in agreement with those found from AFM and DLS analyses (Figure 3a–c) corresponding to 2D MoS_2_, nanometer-sized globules from carbon black as well as nanotubes were found. Li metal foil was used as both the counter and reference electrode, and 1 M LiPF_6_ solution in an ethylene carbonate/diethylene carbonate solvent mixture (1/1 weight ratio) as the electrolyte (see the Section 2 for details). Figure 5a and Figure 5b show the first four CVs of the ee-MoS_2_ and lpe-MoS_2_ samples, respectively, at a potential scan rate of 0.2 mV s^−1^. All the voltages are referenced to Li/Li^+^, i.e., given as V vs. Li/Li^+^, but will be henceforth given just as V for simplicity. The electrodes were biased from the OCV to 3.00 V (average OCV of 2.60–3.00 V after coin cell assembling) and then towards cathodic potentials. An initial comparison between lpe-MoS_2_ and ee-MoS_2_ revealed a strong qualitative resemblance, although the observed peaks tended to be more intense for ee-MoS_2_, suggesting a higher extension of the Li storage processes for the latter.

The lithium storage mechanism in MoS_2_-based electrodes is thought to involve both intercalation and conversion processes during discharge, whereas the opposite processes take place during charge [9]. During the first discharge (black trace in Figure 5a,b), two prominent peaks emerged at 0.99 and 0.44 V for ee-MoS_2_, and 1.00 and 0.41 V for lpe-MoS_2_, with a soft split at ~0.60 V in both cases. The peak at ~1.00 V is ascribed to Li^+^ intercalation into the MoS_2_ lattice according to Equation (3) [9,49]:

MoS_2_ + x Li^+^ + xe^−^ → Li_x_MoS_2_ (0 ≤ x ≤ 1), (3)

which results in a phase transition from semiconducting 2H to semimetallic 1T MoS_2_ for x > 0.29–0.35 [41]. The peaks located at 0.41–0.44 V with a soft split at ~0.60 V are consistent with the irreversible conversion of 1T Li_x_MoS_2_ to metallic Mo nanoparticles and Li_2_S [9,49]:

1T Li_x_MoS_2_ + (4 − x) Li^+^ + (4 − x) e^−^ → Mo + 2 Li_2_S, (4)

where the split at ~0.60 V suggested a two-step conversion mechanism. In addition, the formation of the solid-electrolyte interphase (SEI) is thought to occur during the first cathodic scan at potentials that overlap with those of the Li_x_MoS_2_ conversion reaction at 0.40–0.60 V [50,51]. During charge, both MoS_2_ materials exhibited a weak peak at ~1.70 V (O_2_ peak) that was slightly more intense for lpe-MoS_2_ (see insets to Figure 5a,b). Such a peak has been attributed to the formation of soluble lithium polysulfides (Li-PSs; Li_2_S_n_, 4 ≤ n ≤ 8) from the oxidation of Li_2_S [52,53,54], although other reports suggest two-step oxidation of Mo nanoclusters to give Mo^4+^ (~1.47 V) and Mo^6+^ (~1.70 V) [55,56], as well as partial delithiation of unreacted 1T Li_x_MoS_2_ [7,56]. However, the most prominent peak of the anodic scan appeared at ~2.30 V (O_1_ peak), ascribed to the oxidation of Mo nanoparticles back to (amorphous) 2H-MoS_2_ [Equation (5)] [7,56,57] and Li_2_S oxidation to elemental S [S_8_, Equation (6)] and Li-PSs [9,18,54,58].

Mo + 2 Li_2_S → 2H-MoS_2_ + x Li^+^ + x e^−^ (5)

8 Li_2_S → S_8_ + 16 Li^+^ + 16 e^−^ (6)

In the second discharge, the peaks associated with Li^+^ intercalation and Li_x_MoS_2_ conversion noticed in the first discharge tended to vanish and to shift slightly (peaks denoted as R_2_ and R_3_; see Figure 5a,b, red traces). Further, a new peak (R_1_) appeared at 1.91–1.93 V with a shoulder at ~2.10 V, which shifted to 1.88 V after four cycles. Peaks R_1_ and R_2_ were ascribed to the reduction of elemental S back to Li_2_S [reverse of Equation (6)] and Li-PSs [9,54,59], respectively, although R_2_ could also arise from some lithiation of restored 2H MoS_2_ [7,55,56]. In line with the latter, the weak R_3_ feature would correspond to the conversion of some remaining Li_x_MoS_2_ material [9,58]. In the second charge cycle, both MoS_2_ electrodes exhibited a weak feature at ~1.70 V (O_2_ peak; reactions involving Li-PSs) together with an intense peak at 2.32–2.34 V (O_1_ peak). All these features then appeared recurrently with little changes in the subsequent CV scans (green and blue traces in Figure 5a,b). We, therefore, conclude that lithium storage in both the ee-MoS_2_ and lpe-MoS_2_ electrodes relies on intercalation and conversion reactions, with Li_2_S/S_8_ conversion processes very likely playing a main role. Here, the presence of Mo nanoclusters/nanoparticles is thought to be beneficial, as this metal is known to be efficient at immobilizing Li-PSs as well as catalyzing their conversion reactions, thus contributing to alleviating the shuttle issues associated with the Li_2_S/S_8_ conversion processes [53,54,60].

**Figure 6:**
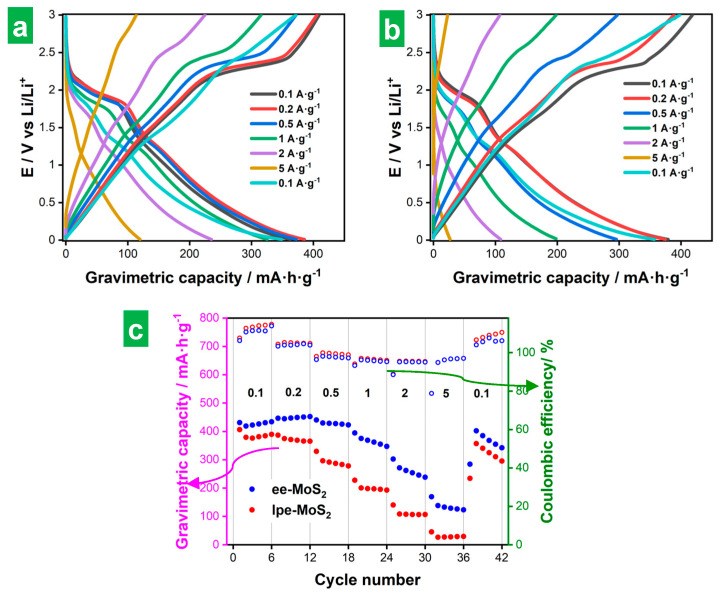
Galvanostatic charge–discharge (GCD) of the exfoliated MoS_2_ electrodes. Charge/discharge profiles at several gravimetric currents for (**a**) ee-MoS_2_ and (**b**) lpe-MoS_2_ electrodes. (**c**) Gravimetric discharge capacities (full circles) and Coulombic efficiency values (hollow circles) for ee-MoS_2_ (blue) and lpe-MoS_2_ (red) at different gravimetric currents (in A/g units). The gravimetric capacities are calculated relative to the total mass of the electrode.

Figure 6a and Figure 6b show representative GCD profiles for ee-MoS_2_ and lpe-MoS_2_ electrodes, respectively, measured at different currents. Specifically, each profile is the fifth of five cycles recorded at the same current. They were recorded after the CVs shown in Figure 5 and that is why the capacity associated with SEI formation is absent from them. The corresponding gravimetric capacities for the five GCD cycles (relative to the total mass of the electrode) are plotted in Figure 6c, together with their coulombic efficiency values. As expected, the profiles displayed clear potential plateaus at low currents (e.g., 0.1 and 0.2 A g^−1^) reflecting the Li_2_S/S_8_ conversion processes at ~2.1–2.3 V vs. Li/Li^+^, although such plateaus were only retained in part at higher currents. Indeed, the shape of the profiles tended to become straight at the highest currents (up to 5 A g^−1^), such constant variation of potential vs. time indicating increasing contributions of (pseudo)capacitive processes [54,61]. The straight, steep nature of the profiles at increasing currents was also indicative of ohmic polarization, which resulted in capacity losses and was more pronounced for lpe-MoS_2_ [54]. Ohmic polarization was also reflected in the GCD profiles by a sudden decrease in potential when the sign of the intensity was inverted. When the current density was returned to its starting low value (0.1 A g^−1^; cyan traces in Figure 6a,b), only a partial recovery of the potential plateaus was observed, implying some irreversibility of the corresponding storage processes. Nonetheless, the total capacity was very similar to that of the first 0.1 A g^−1^ routine (compare cyan and black GCD profiles). In any case, the shape of the GCD profiles (Figure 6a,b) and the capacity values (Figure 6c) were better preserved at higher currents with the ee-MoS_2_ electrode, implying a better overall electrochemical performance of this material compared to its lpe-MoS_2_ counterpart. In particular, the ee-MoS_2_ electrode boasted larger gravimetric capacities at all currents between 0.1 and 5 A g^−1^ as well as a higher rate capability than those of lpe-MoS_2_ (30% vs. 8% capacity retained when the current density was increased from 0.2 A·g^−1^ to 5 A·g^−1^). The values of the coulombic efficiency were in general quite similar for both electrodes. The gravimetric capacity of both exfoliated MoS_2_ materials compared favorably with the theoretical capacity of graphite (372 mA·h·g^−1^), the commercial anode material par excellence for LIBs, showing values of 47 and 371 mA·h·g^−1^ at 0.2 A·g^−1^ for ee-MoS_2_ and lpe-MoS_2_, respectively (Figure 6c). As mentioned above, these gravimetric capacity values were calculated relative to the total mass of the electrode but, if they were calculated relative to the mass of active material, the comparison would be even more favorable, reaching values close to or even higher than the theoretical capacity of MoS_2_. The latter has been explained in the literature as coming from the contribution of capacitive processes derived from the nanostructuring of MoS_2_ [7,9]. These capacity values show the potential of the exfoliated materials, especially that of ee-MoS_2_. Although its rate capability (and cyclability, as will be seen below) is limited, we expect that these parameters could be improved by optimization of the electrode formulation or, as recent reports suggest, through combination with materials with high electrical conductivity [7,9].

**Figure 7:**
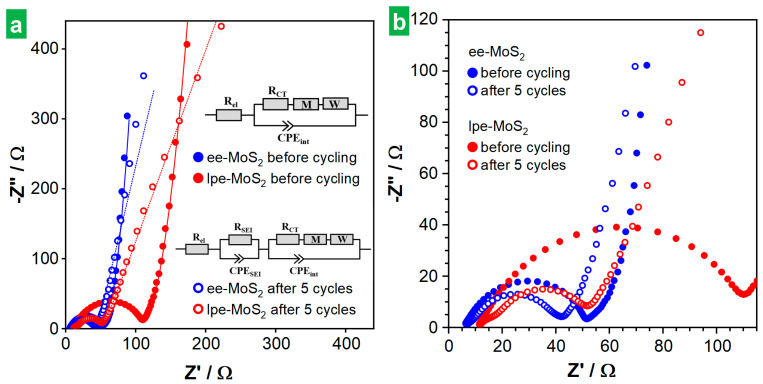
Electrochemical impedance spectroscopy (EIS) of the exfoliated MoS_2_ materials. (**a**) Nyquist plot after 4 h at the OCV (full circles) and after five charge/discharges cycles (hollow circles) for ee-MoS_2_ (blue) and lpe-MoS_2_ materials (red). Their fitting to the indicated equivalent circuits are graphed with solid and dotted lines, respectively. (**b**) Magnification of the high-frequency region of the Nyquist plots in (**a**).

The behavior of the MoS_2_-based electrodes was also investigated by EIS. Figure 7a,b show Nyquist plots of the ee-MoS_2_ and lpe-MoS_2_ electrodes after 4 h of resting at the open circuit voltage (OCV) before any electrochemical measurement (solid traces) and after recording 5 full CVs (hollow traces). A magnification of the high-frequency region of the plots is provided in Figure 7b. EIS was modeled using different electrical equivalent circuits for the electrodes. Before cycling, a model R_el_(R_CT_WM)(CPE_int_) was used [7], where R_el_ is the sum of the resistance of the electrolyte, separator and internal resistance of the cell. A parallel circuit (R_CT_WM)(CPE_int_) simulated the charge storage activity at the electrode–electrolyte interface, where R_CT_ is the charge transfer resistance and CPE_int_ is the capacitance at the interface. The latter is represented by a constant phase element (CPE) to account for its frequency-dispersed behavior. W and M are both open circuit termini accounting for diffusion processes, with W as the Warburg element, modeling semi-infinite linear unrestricted diffusion to a large planar electrode, while M accounts for finite length restricted diffusion. To model the EIS of the electrodes after 5 cycles, a parallel circuit (R_SEI_) (CPE_SEI_) was added to account for the contribution of the SEI, where R_SEI_ and CPE_SEI_ are the SEI resistance and capacitance (also a CPE), respectively [7]. The initial electrode resistance (R_el_), related to the intercept of the plot with the Z’ axis at the highest frequency, was seen to be lower for ee-MoS_2_ (6.8 Ω) than it was for lpe-MoS_2_ (11.4 Ω). Such a difference can in principle be put down to the thinner nature of the ee-MoS_2_ NSs: thinner 2D objects are expected to be more flexible than thicker ones, and hence contacts between neighboring NSs in the electrode should be more conformal, leading to a lower inter-nanosheet resistance [62]. The charge transfer resistance (R_CT_), associated with the diameter of the semicircle in the Nyquist plots, was also smaller with ee-MoS_2_ (45 vs. 139 Ω). The better charge transfer kinetics of this material can be related again to its thinner nanosheets, as it implies a larger active material–electrolyte contact area [63,64]. After recording the CVs, the Nyquist plots revealed a small increase in R_el_ (<1 Ω for both electrodes), ascribed to SEI formation. On the other hand, the R_CT_ became smaller in both cases (35 and 47 Ω for ee-MoS_2_ and lpe-MoS_2_, respectively), which could be attributed to morphological changes taking place in the active material upon cycling, as will be discussed below, e.g., to the development of nanoporosity on the MoS_2_ surface [7]. Likewise, the almost vertical straight-line characteristic of the low-frequency region of the initial Nyquist plot for both electrodes was largely retained after recording the CVs with ee-MoS_2_, but not with lpe-MoS_2_, which became slanted. The latter was indicative of more diffusion-controlled behavior that at least in part accounted for the poorer electrochemical performance of this electrode.

**Figure 8:**
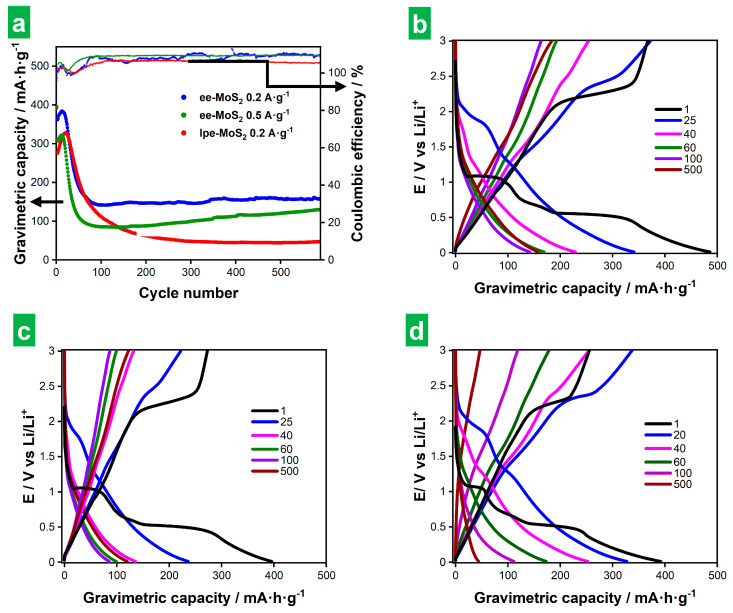
Long-term cyclability of ee-MoS_2_ and lpe-MoS_2_ electrodes on the basis of GCD measurements. (**a**) Gravimetric capacity (larger circles) and Coulombic efficiency (smaller circles) vs. cycle number for ee-MoS_2_ at 0.2 (blue color) and 0.5 A g^−1^ (green), and for lpe-MoS_2_ at 0.2 A g^−1^ (red). (**b**–**d**) GCD profiles recorded at different cycle numbers (indicated in the legends) for ee-MoS_2_ at (**b**) 0.2 A g^−1^ and (**c**) 0.5 A g^−1^ as well as for lpe-MoS_2_ at (**d**) 0.2 A g^−1^. The gravimetric capacities are calculated relative to the total mass of the electrode.

Figure 8a shows results on the long-term GCD cycling at 0.2 and 0.5 A g^−1^ for ee-MoS_2_ and 0.2 A g^−1^ for lpe-MoS_2_, with profiles at selected cycles shown in Figure 8b–d. SEI formation and other processes that took place mainly during the first cycle, as discussed above for the CVs (see Figure 5 and accompanying text), showed up in the first cycle in the form of marked plateaus. However, as the number of GCD cycles progressed into a few or several tens, a clear fading of the voltage plateaus, even those corresponding to Li_2_S/S_8_ conversion processes at 2.1–2.3 V, was observed. Indeed, the CVs after long-term cycling (see Figure S4a,b in Section S2 of the Supplementary Materials) suggested that the conversion reactions taking place in the bulk of the active material were no longer the major contributors to electrode capacitance and that surface-controlled processes became dominant instead. Such a decline in conversion reactions could stem from structural changes affecting the active material during cycling or the irreversible exhaustion of 2H-MoS_2_ [7,61,65]. As shown in Figure 8a, the capacity increased somewhat during the first 10–20 GCD cycles for all the tested electrodes and then decreased rapidly over a few (ee-MoS_2_) or several (lpe-MoS_2_) tens of additional cycles. We note that the first cycle, where SEI formation took place, afforded an initial high capacity that is not considered in this discussion. Afterward, a stable cycling behavior was observed, during which the measured capacity tended to remain constant (lpe-MoS_2_; ~45 mAh g^−1^) or to increase somewhat (ee-MoS_2_, especially at 0.5 A g^−1^; in the 100–200 mAh g^−1^ range). The capacity values in the stable cycling region were much larger with the ee-MoS_2_ electrode than they were with their lpe-MoS_2_ counterpart. Thus, the thinner nature of the former has been shown to be beneficial to its global electrochemical performance, both to its capacity and rate capability (Figure 6c) and to its cycle life (Figure 8). The Coulombic efficiency fell below 100% during the first cycles due to the SEI formation and then increased to values higher than 100% for all the electrodes, the effect being more pronounced for the ee-MoS_2_-based electrode than for the lpe-MoS_2_-based one. The origin of Coulombic efficiencies larger than 100% is not clear at the moment. However, their occurrence has been previously reported in the literature [7,11,66] and attributed to surface-based oxidation processes developed upon cycling [7]. Modeling of the EIS after long-term cycling (Figure S4c in Section S2 of the Supplementary Materials) showed that the SEI became best simulated by a parallel circuit (R_SEI_) (C_SEI_) where R_SEI_ and C_SEI_ are the SEI resistance and capacity, respectively [7].

The type of cycling behavior observed here with the MoS_2_-based electrodes has been previously described [10]. It was demonstrated that the different cycling regimes (i.e., capacity increase, decrease, stabilization) result from morphological changes occurring at the electrode. These changes involve alterations in the exposed surface area, electrolyte permeability or electrical conductivity, which affect the storage capacity [7,65]. For example, the increase in capacity during the first few tens of cycles was explained by the development of a nanopore-rich surface morphology upon cycling [7]. Such an opening of nanoporosity promoted electrolyte penetration and contact with the active material, thus improving lithiation, and leading to an activation process of the electrode [67]. The fact that fewer cycles were required to reach the top capacity in this first regime with the ee-MoS_2_ electrode, relative to the case of lpe-MoS_2_, suggested a more efficient evolution of the surface morphology in the former material. In turn, this was probably due to a larger exposed surface area of the ee-MoS_2_ NSs arising from their thinner nature [10,67]. Concerning the capacity decrease regime, it is known that MoS_2_-based electrodes shift from a battery-type storage mechanism to extrinsic pseudocapacitive behavior with an increasing nanostructuring of the material [68,69]. Thereby, it could be argued that the capacity decay during this regime was rooted in the variable contributions from decreasing battery-like reactions and emerging pseudocapacitive processes that stemmed from the morphological changes experienced by the active material. Such a trend towards less battery-like and more pseudocapacitive behaviors was clearly observed in the GCD profiles of Figure 8b–d. The third regime (capacity stabilization) would then be reached when charge storage becomes completely dominated by pseudocapacitive processes and any purely battery-type behavior is rendered largely residual. Here, the much larger capacity values afforded by the ee-MoS_2_ electrode could again well be the result of the thinner nature of its corresponding NSs. Specifically, when morphological and compositional changes are triggered in the MoS_2_ material by the cycling routine, we would expect the resulting primary active particles to be smaller if they originate from thinner NSs. In turn, smaller active particles should favor the surface-driven, pseudocapacitive processes that dominate the third regime and thus lead to larger capacities. This reasoning would also explain the larger number of cycles required for the lpe-MoS_2_ electrode to reach the third regime, as thicker MoS_2_ NSs would take longer to fully convert to the final active products. Post-mortem microscopic studies of the cycled electrodes confirmed these expectations and showed their thorough morphological transformation after long-term cycling. Indeed, the original morphology (see Figure S5 in Section S3 of the Supplementary Materials) changed completely, displaying microlamellae (Figure S6a,b for ee-MoS_2_, Figure S8a,d for lpe-MoS_2_), microspheres (Figures S6c and S7c,d for ee-MoS_2_, Figure S8c for lpe-MoS_2_), as well as combinations of both (Figure S6d), and other morphologies (Figure S8b,e,f). For the electrodes cycled at 0.2 A g^−1^, the microspheres were smaller and more abundant for the electrodes based on ee-MoS_2_ than for those based on lpe-MoS_2_ (Figure 6c,d vs. Figure 8c), which implies a higher surface area. As for the microlamellae, their surface showed more rugosity in the case of ee-MoS_2_ than in that of lpe-MoS_2_ (Figure S6e,f,h,i vs. Figure S8d). The more developed surface in ee-MoS_2_-based electrodes would explain their higher gravimetric capacity after cycling (Figure 8a), given that the lithium storage is dominated by surface-driven, pseudocapacitive processes in this regime. In the case of lpe-MoS_2_ electrodes, a minoritarian nanoflower-like morphology, similar to that developed by solvothermal MoS_2_ [18], which could contribute to some extent to the surface area, was also detected (Figure S8b,e,f). The ee-MoS_2_-based electrode cycled at 0.5 A g^−1^ also displayed microlamellae and microspheres (Figure S7), but the most frequent morphology showed the development of porosity (Figure S7a,b), which is indicative of an activation process. The shortening of the ion transport paths could explain the increasing trend of the capacity of this electrode upon cycling (Figure 8a, green circles).

**Figure 9:**
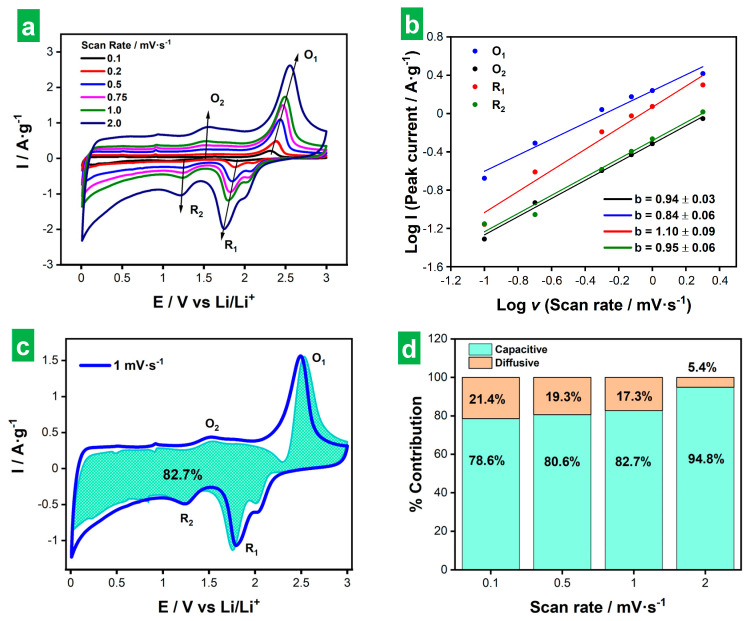
Kinetic analysis of the ee-MoS_2_ electrode. (**a**) CVs recorded at several scan rates between 0.1 and 2 mV s^−1^. (**b**) Determination of *b* values for the main redox peaks shown in (**a**). (**c**) Representation of (pseudo)capacitive contribution (green-shaded plot) to the CV recorded at 1 mV s^−1^ (blue plot). (**d**) Contributions from (pseudo)capacitive and diffusion-controlled processes to the total capacity of the electrode measured at different potential scan rates.

Finally, an analysis of the kinetic behavior of the ee-MoS_2_ electrode was carried out to probe the nature of its charge storage mechanism. To this end, CVs were recorded at different potential scan rates between 0.1 and 2 mV s^−1^ (Figure 9a). The main redox peaks (i.e., O_1_, R_1_, O_2_, R_2_, as previously defined in Figure 5) were then fitted to the following exponential equation to determine the values of the exponent (*b*)

*i* = *aν^b^*, (7)

where *i* is the measured peak current at a given scan rate, *ν*, and *a* and *b* are adjustable parameters. A *b* value of 1 denotes purely (pseudo)capacitive charge storage processes that are not limited by diffusion, while a value of 0.5 indicates purely diffusion-controlled processes. *b* values in between these two extremes imply the occurrence of mixed (pseudo)capacitive and diffusion-controlled processes in different proportions [70]. The results of peak fitting are presented in Figure 9b. For the O_1_/R_1_ redox pair, the *b* parameters were calculated as 0.84 (O_1_) and 1.10 (R_1_). The latter was obviously artifactual and very likely stemmed from contributions of the shoulder peak observed in Figure 9a at the positive potential side of the R_1_ peak (i.e., in the 2.0–2.1 V region). The separation between the R_1_ and shoulder peaks tended to decrease with increasing *ν*, implying an increasingly large contribution of the shoulder peak to the R_1_ current and thus enhancing its *b* value. For the O_2_/R_2_ pair, the *b* values were 0.94 (O_2_) and 0.95 (R_2_). All these figures indicated charge storage in the ee-MoS_2_ electrode to comprise both (pseudo)capacitive and diffusion-limited processes, but with a very clear dominance of the former. Such a conclusion was reasonable, considering that these redox peaks were associated with Li_2_S/S_8_ and Li-PS conversion reactions, which are thought to be mainly surface processes and are consequently not or little affected by diffusion [53,54,71].

To further discriminate the (pseudo)capacitive and diffusion-controlled contributions to the measured current in the CVs, the following equation was employed for fitting routines

*i* = *k_1_ν* + *k_2_ν*^1/2^, (8)

where the first (second) term on the right-hand side of the equation represents the (pseudo)capacitive (diffusion-controlled) contribution to the total measured current, and *k_1_* and *k_2_* are adjustable parameters [70,72]. Figure 9c shows the actual CV recorded at 1 mV s^−1^ together with its (pseudo)capacitive contribution as derived from Equation (8). In agreement with the results of the fitting of *b* parameters, (pseudo)capacitive processes totally dominated the charge storage behavior (82.7% of the total capacity). Figure 9d displays the contributions from (pseudo)capacitive and diffusion-controlled processes to the total capacity of the electrode measured at different potential scan rates. As expected for any material and previously found for this particular one from the results in Figure 6a, the contribution of (pseudo)capacitive processes to the total capacity increased with increasing potential scan rate. However, for the present ee-MoS_2_ material, this contribution was already quite high at low rates (e.g., 78.6% at 0.1 mV s^−1^). The latter was in contrast with typical results from MoS_2_-based electrodes, which tend to exhibit lower contributions at low scan rates [71,73,74,75]. Again, this particular behavior of ee-MoS_2_ could be ascribed to the smaller thickness and hence higher exposed area associated with the ee-MoS_2_ NSs.

## 4. Conclusions

We have demonstrated a simple and straightforward method for the preparation of phase-preserved 2H-MoS_2_ thin nanosheets through cathodic delamination of bulk MoS_2_ crystals with suitable electrolytes. Specifically, trimethylalkylmamonium cations were shown to outperform bulkier tetraalkylammonium with substituents of equal length, which were the subject of previous studies, in accessing thinner nanosheets. On the one hand, the trimethylalkylammonium intercalants were large enough to prevent the intercalation of such a high number of cations that the corresponding electron injection would exceed the 2H to 1T transformation threshold for MoS_2_. On the other hand, their smaller cross-section compared to tetraalkylammonium cations led to lower steric repulsion between the intercalated areas, promoting a more homogeneous and smoother intercalation that allowed obtaining thinner nanosheets after sonication of the expanded crystals. The higher flexibility and smaller thickness of MoS_2_ nanosheets enabled electrode materials with lower electrical resistance and shorter pathways for solid-state diffusion, resulting in higher capacity, rate capability and cycle life as anodes for lithium storage.

## Figures and Tables

**Table 1:** A-band position for MoS_2_ dispersions obtained by cathodic exfoliation using different ammonium salts as electrolytes. Wavelength for the maximum of the A exciton band of the MoS_2_ dispersions obtained by electrochemical exfoliation with different electrolytes at a concentration of 0.05 M in PC using a bias voltage of −8 V.

Electrolyte	λ_A_ (nm)
TMACl	-
TEACl	673.5
TBACl	673.5
THABF_4_	678.0
HTMABr	672.0
TMOABr	676.5
CTAB	676.0

## Data Availability

The raw data supporting the conclusions of this article will be made available by the authors upon request.

## Supplementary Materials

The following supporting information can be downloaded at: https://www.mdpi.com/article/10.3390/nano14110932/s1. Figure S1: microscopic and spectroscopic characterization of ultrasound-assisted liquid-phase exfoliated MoS_2_ colloidal dispersions. Figure S2: AFM characterization of cathodically exfoliated MoS_2_ materials obtained using ammonium salts other than HTMABr as electrolyte. Figure S3: X-ray diffraction patterns of bulk and exfoliated MoS_2_ materials. Figure S4: cyclic voltammograms and electrochemical impedance spectroscopy (EIS) of the exfoliated MoS_2_ materials after long-term cycling. Figure S5: microscopic characterization of the ee-MoS_2_ electrodes. Figure S6: post-mortem microscopic characterization of the ee-MoS_2_ electrodes cycled at 0.2 A g^−1^. Figure S7: post-mortem microscopic characterization of the ee-MoS_2_ electrodes cycled at 0.5 A g^−1^. Figure S8: post-mortem microscopic characterization of the lpe-MoS_2_ electrodes cycled at 0.2 A g^−1^. Supplementary Movies S1–S3: electrochemical expansion of MoS_2_ over 30 min using HTMABr, TEACl and THACl as electrolytes, respectively.
