# Supplementary material for: Two-Dimensional MoS2 Nanosheets Derived from Cathodic Exfoliation for Lithium Storage Applications
Source: Nanomaterials (Basel). 2024 May 25;14(11):932. doi: 10.3390/nano14110932 (PMC11173767; doi:10.3390/nano14110932)
Supplement: Supplementary file 1 [file nanomaterials-14-00932-s001.zip › nanomaterials-2975187-supplementary.pdf]

Supplementary Material

for

Two-Dimensional MoS2 Nanosheets Derived from Cathodic Exfoliation for Lithium Storage Applications

Alberto Martínez-Jódar [1,2], Silvia Villar-Rodil [1,*], José M. Munuera [3,4], Alberto Castro-Muñiz [1], Jonathan N. Coleman [4], Encarnación Raymundo-Piñero [2] and Juan I. Paredes [1,*]

1 Instituto de Ciencia y Tecnología del Carbono, INCAR-CSIC, Francisco Pintado Fe 26, 33011 Oviedo, Spain

2 CEMHTI UPR3079, University of Orléans, CNRS, 1D avenue de la Recherche Scientifique, 45071 Orléans, France

[3] Department of Physics, Faculty of Sciences, University of Oviedo, C/ Leopoldo Calvo Sotelo, 18, 33007 Oviedo, Spain

4 School of Physics, CRANN and AMBER Research Centre, Trinity College Dublin, D02 E8C0 Dublin, Ireland

* Corresponding author: silvia@incar.csic.es (S. Villar-Rodil),

* Corresponding author: paredes@incar.csic.es (J. I. Paredes)

S1. Additional characterization of the exfoliated MoS_2 materials

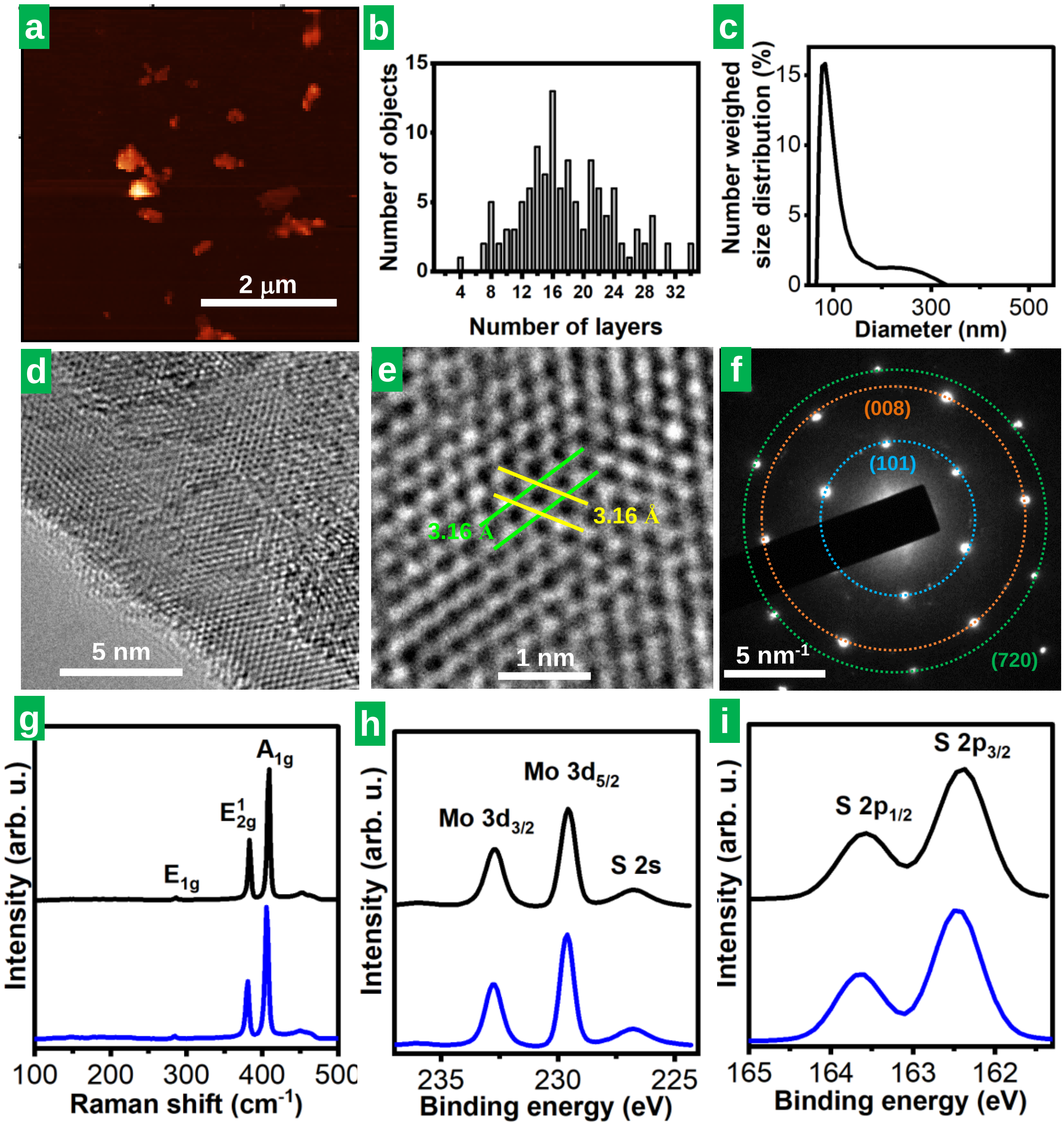

Figure S1. Microscopic and spectroscopic characterization of ultrasound-assisted liquid-phase exfoliated MoS_2 colloidal dispersions. (a) Representative AFM image of the MoS_2 nanosheets deposited onto a Si/SiO_2 substrate from dispersion. **(b)** Histograms of apparent thickness of MoS_2 nanosheets derived from a pool of over 100 nanosheets measured from AFM images. **(c)** DLS-derived number-weighed hydrodynamic diameter distribution for MoS_2 dispersion in DMF. **(d,e)** Representative HR-TEM images of the MoS_2 basal planes at different magnifications. The parallel lines in (e) assist in visualizing *a* and *b* cell parameters in the hexagonal cell of MoS_2 lattice. **(f)** SAED pattern of the MoS_2 lattice with indication of the families of planes involved in the observed diffractions. **(g)** Typical Raman spectra of the starting MoS_2 bulk powder (black trace) and liquid-phase exfoliated MoS_2 (blue trace). **(h, i)** Typical XPS spectra of (h) Mo 3d and (i) S 2p core levels for bulk MoS_2 powder (black trace) and liquid-phase exfoliated (blue trace) MoS_2 NSs. The main bands have been labeled for clarity.

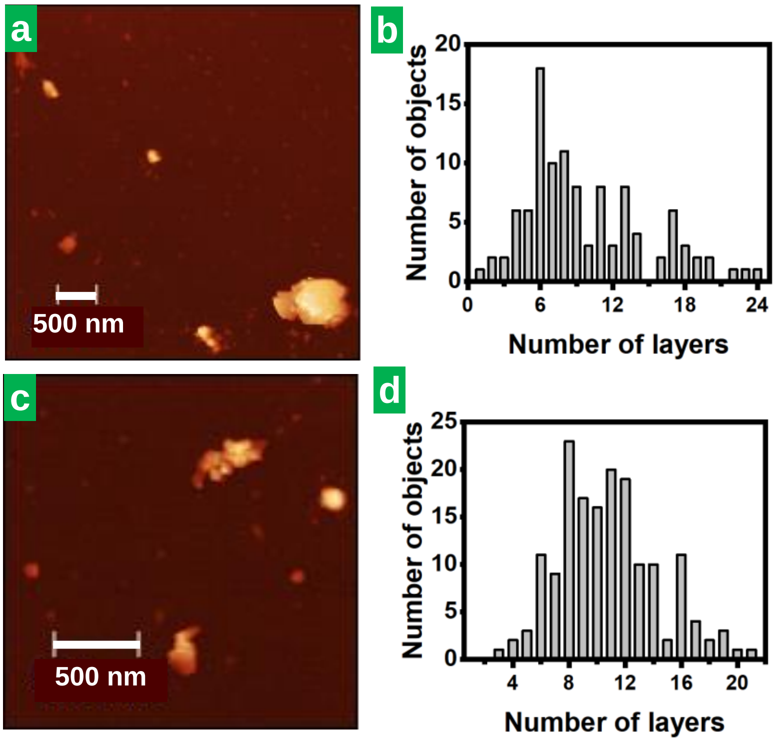

Figure S2. AFM characterization of cathodically exfoliated MoS_2 materials obtained using ammonium salts other than HTMABr as electrolyte. Representative AFM images of the MoS_2 nanosheets deposited onto a Si/SiO_2 substrate from dispersions prepared using **(a)** $HTHABF_4$ and **(c)** TMOABr as electrolytes. Histograms of apparent thickness of MoS_2 nanosheets derived from a pool of over 100 nanosheets measured from AFM images for materials obtained using **(b)** $HTHABF_4$ and **(d)** TMOABr as electrolytes.

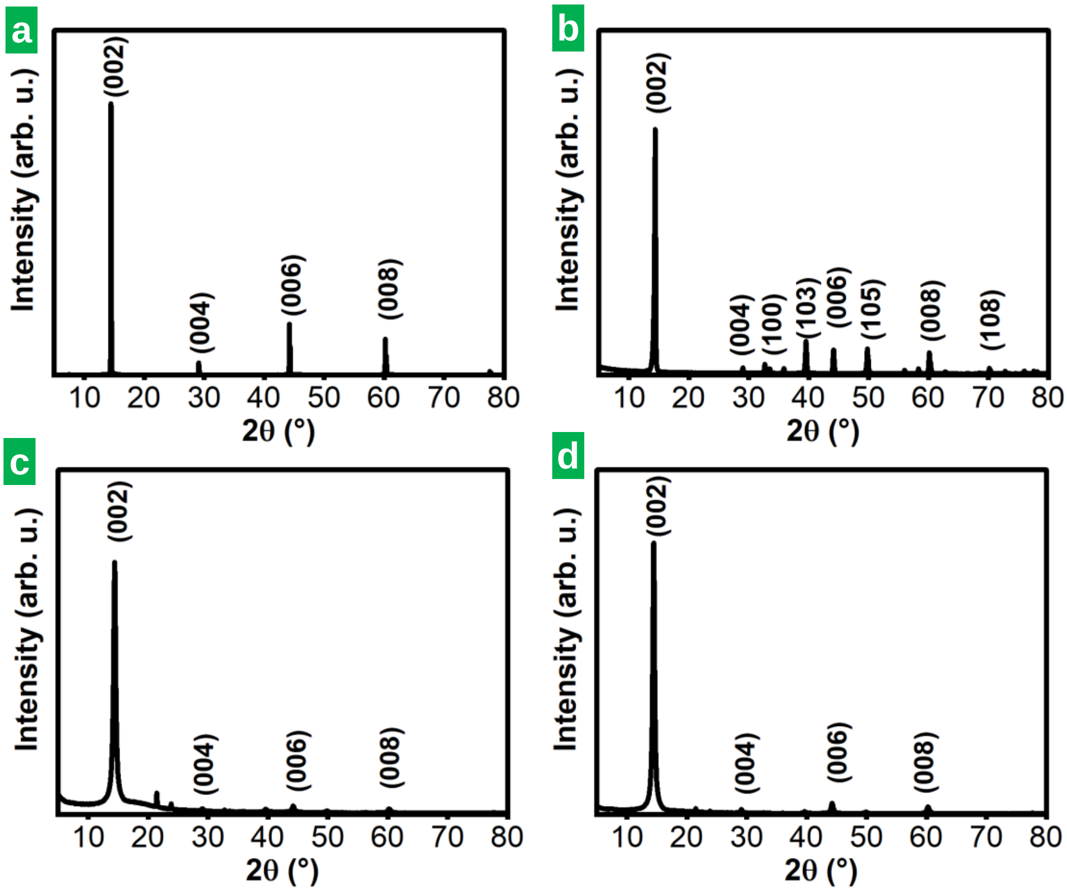

Figure S3. X-ray diffraction (XRD) patterns of bulk and exfoliated MoS_2 materials. X-ray diffraction patterns of both MoS_2 parent materials, namely, **(a)** bulk crystal and **(b)** bulk powder, as well as of films prepared from dispersions of exfoliated materials derived from them: **(c)** electrochemically exfoliated and **(d)** liquid-phase exfoliated MoS_2 nanosheets, respectively. The XRD peaks have been labelled according to JCPDS card 37-1492.

S2. Additional electrochemical characterization of the electrodes based on exfoliated MoS_2

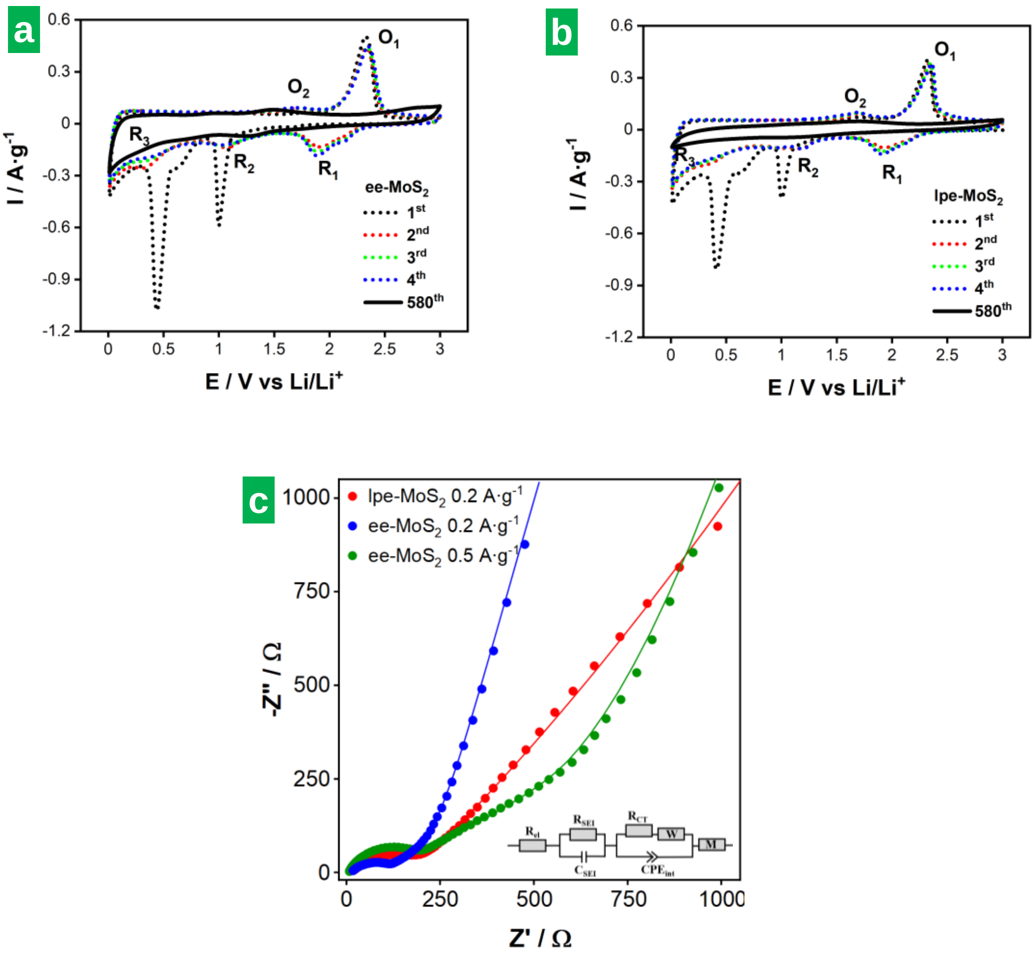

Figure S4. Cyclic voltammograms (CVs) and Electrochemical impedance spectroscopy (EIS) of the exfoliated MoS_2 materials after long-term cycling. CVs of the first four (dotted trace) and after long-term cycling (solid trace) of **(a)** ee-MoS_2 and **(b)** lpe-MoS_2 electrodes. Scan rate: 0.2 mV s^{-1}. **(c)** EIS Nyquist plots after the long-term cycling for ee-MoS_2 electrode at 0.2 A g^{-1} (blue circles) and 0.5 A g^{-1} (green circles), and for lpe-MoS_2 electrode at 0.2 A g^{-1} (red circles). The fitting of the experimental data to the indicated electrical equivalent circuit used for data modelling is shown in solid traces of the same color as the corresponding experimental data.

S3. Post-mortem studies of the electrodes based on exfoliated MoS_2

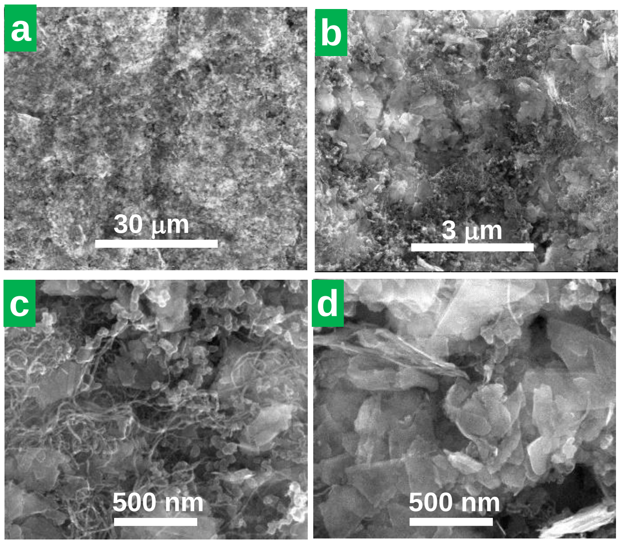

Figure S5. Microscopic characterization of the ee-MoS_2 electrodes. FE-SEM images of the starting ee-MoS_2 electrode at different magnifications.

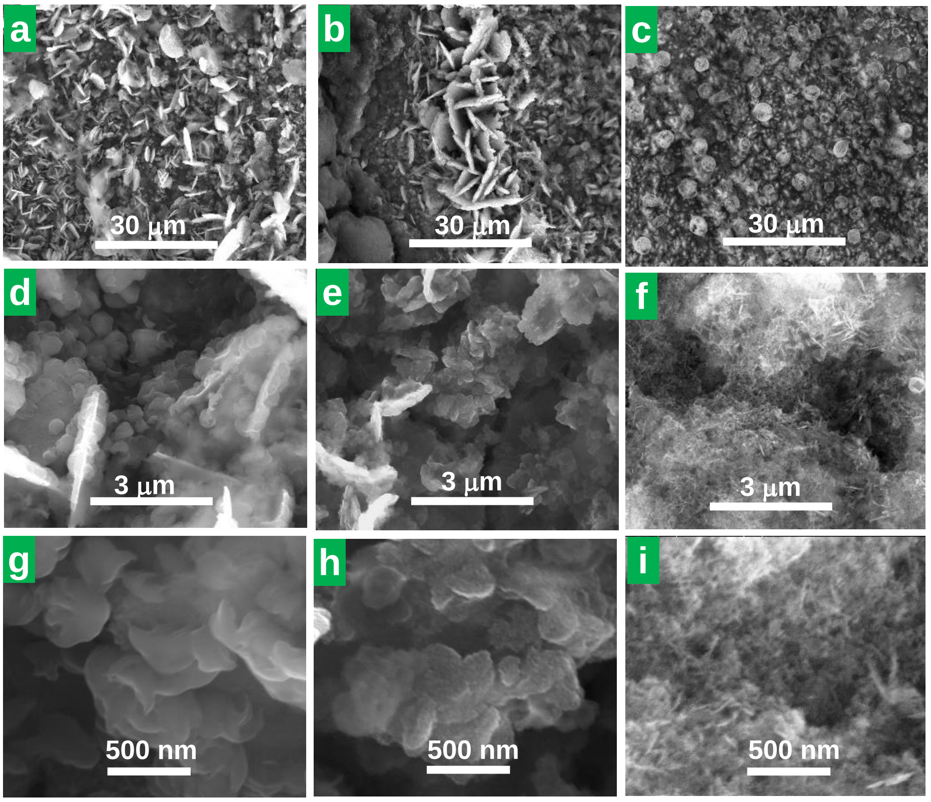

Figure S6. Post-mortem microscopic characterization of the ee-MoS_2 electrodes cycled at 0.2 A g^{-1}. FE-SEM images of the ee-MoS_2 electrodes after long-term cycling at 0.2 A g^{-1} showing varied morphologies at different magnifications.

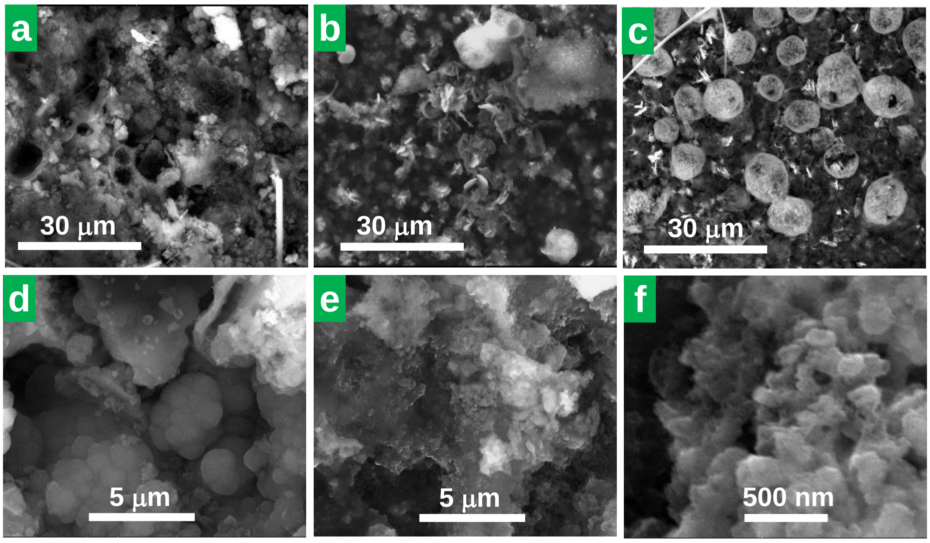

Figure S7. Post-mortem microscopic characterization of the ee-MoS_2 electrodes cycled at 0.5 A g^{-1}. FE-SEM images of the ee-MoS_2 electrodes after long-term cycling at 0.5 A g^{-1} showing varied morphologies at different magnifications.

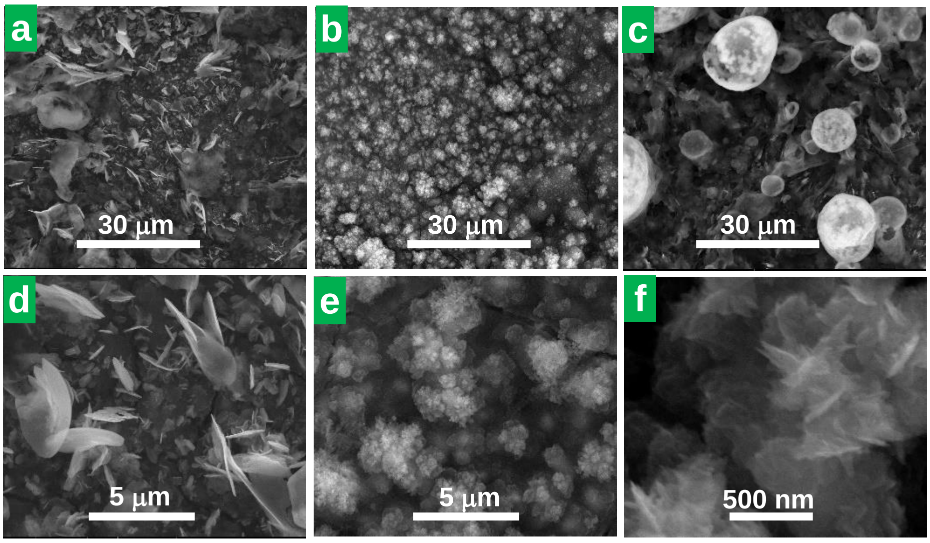

Figure S8. Post-mortem microscopic characterization of the lpe-MoS_2 electrodes cycled at 0.2 A g^{-1}. FE-SEM images of the lpe-MoS_2 electrodes after long-term cycling at 0.2 A g^{-1}, showing varied morphologies at different magnifications, namely: **(a,d)** microlamellae, **(c)** microspheres and **(b,e,f)** nanoflower-like microstructures.
